# A meta-analysis of unemployment risk factors for middle-aged workers

**DOI:** 10.5271/sjweh.4216

**Published:** 2025-05-01

**Authors:** Rahman Shiri, Joonas Poutanen, Mikko Härmä, Jenni Ervasti, Eija Haukka

**Affiliations:** 1 Finnish Institute of Occupational Health, Helsinki, Finland.

**Keywords:** depression, employment, exercise, job control, mental health

## Abstract

**Objective:**

This systematic review and meta-analysis aimed to identify risk factors for unemployment among middle-aged workers.

**Methods:**

Searches were carried out in PubMed, Web of Science and Google Scholar until November 2024, focusing on observational longitudinal studies that involved workers aged 40–64 years. Three reviewers evaluated the quality of the studies. A random-effects meta-analysis was employed, and heterogeneity and publication bias were assessed.

**Results:**

Out of 10 432 reports, 19 longitudinal studies (N=374 585 participants) were included in the review. The meta-analysis identified multiple risk factors associated with unemployment, including suboptimal self-rated general health [hazard ratio (HR) 1.44, 95% confidence interval (CI) 1.21–1.70], mental health conditions [HR 1.48, 95% CI 1.30–1.68, particularly depressive symptoms (HR 1.42, 95% CI 1.14–1.76)], low job control (HR 1.30, 95% CI 1.17–1.43), and lack of physical activity (HR 1.56, 95% CI 1.13–2.15). Additionally, a higher risk of unemployment was observed among individuals with ≤12 years of education (HR 1.17, 95% CI 1.00–1.36), those who are unmarried, separated, or widowed (HR 1.43, 95% CI 1.14–1.79), and immigrants (HR 1.26, 95% CI 1.11–1.44). Age, gender, smoking, alcohol consumption, musculoskeletal disorders, cardiovascular and respiratory diseases, digestive diseases, diabetes, neoplasm, and limitations in daily activities did not increase unemployment risk.

**Conclusions:**

This meta-analysis highlights the impact of mental health conditions, low job control, and lack of leisure-time physical activity on unemployment risk among middle-aged workers. Interventions aimed at improving mental health and increasing job control and physical activity could reduce unemployment risk.

In 2023, the global unemployment rate reached 5.1%, with low-income countries experiencing a slightly higher rate of 5.7%, compared to 4.5% in high-income countries (1). Within the European Union, the rate stood at 6.1% among the population aged 15–74 years (2). Unemployment carries many negative consequences for individuals and incurs substantial societal costs. On a personal level, lack of employment is often associated with a decline in both mental and physical health (3–5). Furthermore, it significantly increases the risk of social and economic hardships (6).

There is a need to boost workforce participation and productivity. As the working population ages and labor demands grow, it becomes increasing necessary to prolong working life durations (7). This demographic shift results in a workforce that increasingly consists of mid-career and senior employees (8). A significant challenge associated with the growing number of elderly workers is the accelerated decline in work capacity and ability after the age of 50 (9, 10). Identifying older individuals who are at an increased risk of unemployment is vital for developing and implementing preventative strategies. These individuals are an integral and growing part of the labor force. By addressing the risk factors of unemployment, we can ensure that older workers remain productive and engaged in the workforce for longer periods.

Previous research has identified several factors associated with higher unemployment risk. These include a lack of physical activity (11), low job control (11), poor self-rated general health (12–14), mental health conditions (13–17), comorbidity of three or more chronic physical health conditions (14), insomnia (14) and substance use (18). However, previous studies did not find associations of alcohol use (11, 18) and smoking (11), and unemployment. Notably, problematic alcohol consumption was linked to an increased risk (14, 18). Additionally, research has shown contradictory results for some unemployment risk factors, such as age (13, 19), sex (20, 21), and education (13, 20).

According to the direct selection hypothesis, health directly influences employment. Individuals with poor health are at a higher risk of becoming or remaining unemployed compared to their healthier counterparts (22, 23). Consequently, unemployed individuals with health problems may need additional support to enhance their re-employment prospects (23). While certain risk factors contributing to unemployment have been identified, the specific causes of unemployment among older workers are not well-defined. Therefore, there is a necessity for the consolidation of existing studies to fully comprehend and measure the impact of these risk factors. In the current study, we aimed to conduct a systematic review of the literature examining the associations of sociodemographic, lifestyle, and occupational factors, alongside health conditions with the incidence of unemployment in middle-aged individuals, and to estimate the magnitudes of these associations through meta-analysis.

## Methods

### Search strategy

The PRISMA statement guided the development of our review protocol and meta-analysis (24). Our research involved a comprehensive search of the PubMed and Web of Science databases from the time they were established until November 2024. The search utilized a mix of MeSH terms and text words as outlined in table 1. Furthermore, we expanded our search to include Google Scholar.

**Table 1:** Searches performed on PubMed and Web of Science on 25 November 2024.

Search		Query	No of items found
**PubMed**			
	#1	“Unemployment”[Mesh] OR “disability pension”[tiab] OR “disability retirement”[tiab] OR “early retirement”[tiab] OR “retired early”[tiab] OR “labor market exit”[tiab] OR “labor market exit”[tiab] OR “personnel turnover”[Mesh] OR (quit[tiab] AND job[tiab]) OR (quit[tiab] AND organization[tiab]) OR (quit[tiab] AND organisation[tiab]) OR (quit[tiab] AND employment[tiab])	18 260
	#2	“Middle aged”[Mesh] OR “old people”[tiab] OR “older people”[tiab] OR “old individuals”[tiab] OR “older individuals”[tiab] OR “old participants”[tiab] OR “older participants”[tiab] OR “old workers”[tiab] OR “older workers”[tiab]OR “40 years”[tiab] OR “45 years”[tiab] OR “50 years”[tiab] OR “55 years”[tiab] OR “60 years”[tiab]	5 077 078
	#3	#1 AND #2	6537
	#4	clinical trials and reviews	295
	#5	#3 NOT #4	6242
**Web of Science**			
	#1	ALL=(unemploy*) OR ALL=(“disability pension”) OR ALL=(“disability retirement”) OR ALL=(“early retire*”) OR ALL=(“retired early”) OR ALL=(“labor market exit”) OR ALL=(“labor market exit”) OR ALL=(“personnel turnover”) OR ALL=(quit AND job) OR ALL=(quit AND organization) OR ALL=(quit AND organisation) OR ALL=(quit AND employment)	72 110
	#2	ALL=(“middle aged”) OR ALL=(“middle age”) OR ALL=(“old people”) OR ALL=(“older people”) OR ALL=(“old individuals”) OR ALL=(“older individuals”) OR ALL=(“old participants”) OR ALL=(“older participants”) OR ALL=(“old workers”) OR ALL=(“older workers”) OR ALL=(“40 years”) OR ALL=(“45 years”) OR ALL=(“50 years”) OR ALL=(“55 years”) OR ALL=(“60 years”)	487 523
	#3	#1 AND #2	3354
	#4	reviews, proceeding abstracts, meeting abstracts, letters, and editorials	164
	#5	#3 NOT #4	3190

### Inclusion and exclusion criteria

This review incorporated longitudinal studies targeting middle-aged employees, specifically those aged 45–64. Given that many studies focused on individuals aged ≥40 years, our inclusion criteria were broadened to encompass studies enlisting workers ≥40 years old. These studies were required to investigate a risk factor associated with early old age retirement, disability retirement, unemployment, or job change. In this review we report the results for unemployment only.

This review did not include research that involved clinical subjects, individuals who were exclusively unemployed, solely on sick leave, or only those already retired. Furthermore, we excluded studies that combined various forms of labor market departures into a singular outcome, such as combining disability retirement with unemployment or early old-age retirement with unemployment.

### Quality assessment

Three reviewers independently evaluated the methodological quality of the studies, employing criteria adapted from the Effective Public Health Practice Project quality assessment tool (25). The evaluation focused on five important potential biases: selection, performance, detection, attrition, and confounding, as detailed in the supplementary material (www.sjweh.fi/article/4216) table S1. Any discrepancies between the reviewers were settled through deliberation.

### Meta-analysis

When faced with numerous reports of a single study, we prioritized data extraction from those reports that offered more comprehensive adjustments for confounding variables or encompassed a broader sample size. For each research, we derived the most adjusted risk estimates and their 95% confidence intervals (CI) for the exposures. One study (17) reported a hazard ratio (HR) for females. To obtain the HR for males, we inverted the female HR and utilized the standard error of the female estimate to compute the 95% CI. In assessing the effect of educational attainment on unemployment risk, we recalculated the adjusted HR by contrasting individuals with ≤12 years of education against those with >12 years of education. A fixed-effect meta-analysis was utilized to integrate subgroups within a single study, while a restricted maximum likelihood (REML) random-effects meta-analysis was applied to synthesize the findings from various studies. Meta-regression was employed to investigate the sources of variability in effect size observed across different studies. A funnel plot was employed to investigate publication bias and Egger’s regression test was used to assess the asymmetry of the funnel plot. Additionally, the trim-and-fill method was applied to impute studies potentially missing due to publication bias. For conducting the meta-analyses, Stata version 18 (StataCorp, College Station, TX, USA) was utilized.

## Results

**Figure 1:**
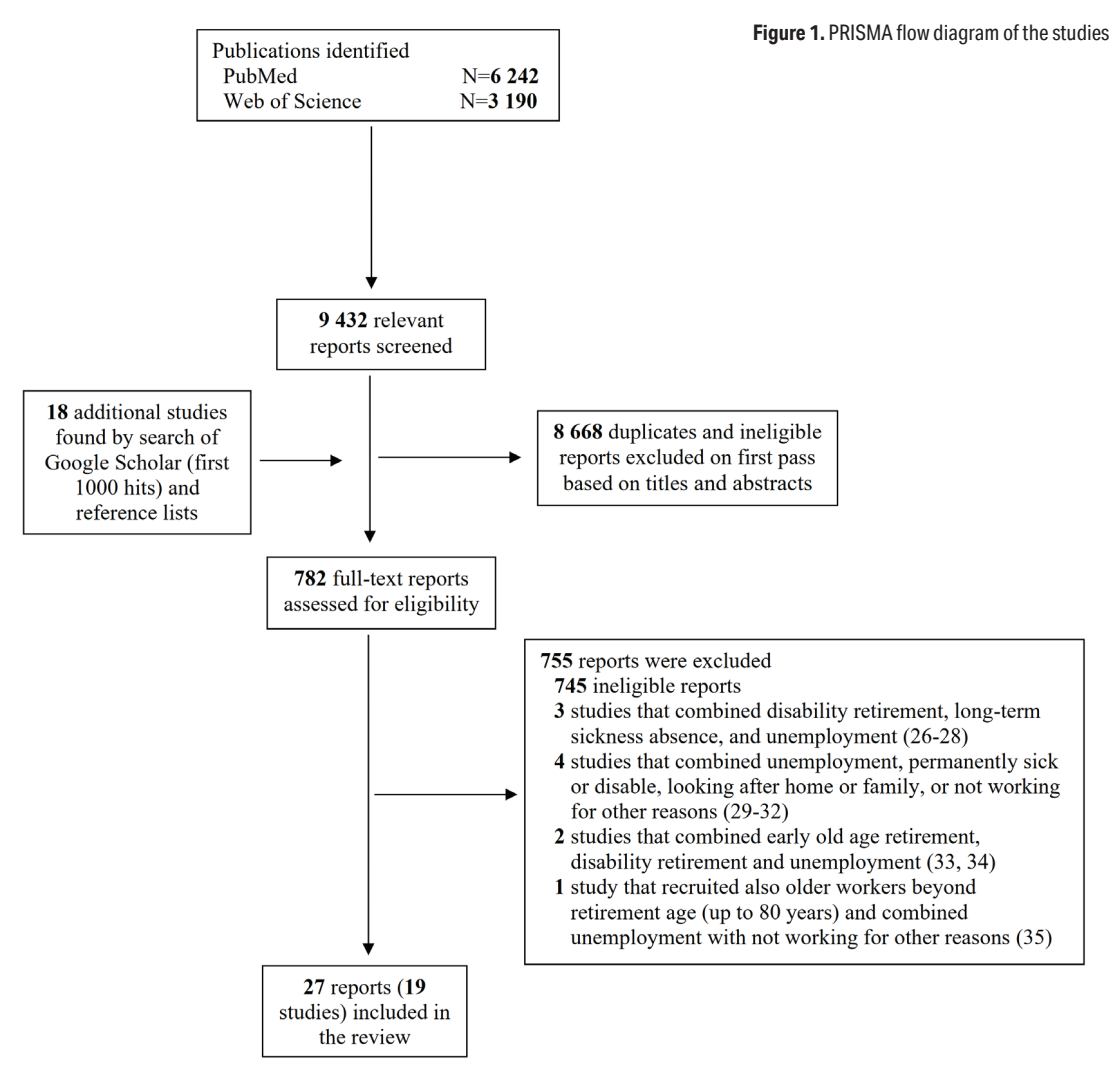
PRISMA flow diagram of the studies selection.

In total, 9432 reports were collected from PubMed and Web of Science, as depicted in figure 1. The primary reviewer conducted the initial screening of titles and abstracts, along with the first 1000 results from Google Scholar, amounting to the total of 10 432. Subsequently, 782 articles were examined in full to assess their relevance. Of these, 755 were dismissed for not studying unemployment among middle-aged individuals and 10 studies (26–35) for failing to satisfy the inclusion criteria. This review ultimately encompassed 27 reports (11, 13, 15–17, 19–21, 36–54), which included 19 longitudinal studies involving 374 585 participants. The results of 6 studies were detailed across 17 distinct reports, while the findings from 4 studies were consolidated into a single report. The number of participants in the studies ranged between 586 and 145 551 individuals (supplementary table S2). The distribution of the studies included 4 each from The Netherlands and Denmark, 3 from Sweden, 2 each from Finland and Germany, and 1 each from Canada, the United Kingdom, and South Korea. There was also a study that included participants from 11 different European countries: Austria, Belgium, Denmark, France, Germany, Greece, Italy, Spain, Sweden, Switzerland, and the Netherlands. Out of the 19 studies examined, 16 were chosen for the meta-analyses, which included 346 158 participants.

### Assessment of methodological quality

In evaluating selection bias, 2 studies received a low-risk rating, 11 were assessed with moderate risk, and 5 were determined to have a high risk, as detailed in supplementary table S3. A particular study, documented in three reports, was assigned a low risk for selection bias concerning one exposure (36) but rated a moderate risk for two additional exposures (37, 38). Regarding performance bias, 1 study earned a low risk, while 16 were deemed moderate risk. For some exposures, 2 studies (3 reports) (36, 38, 39) were considered low risk for some exposures but were given a moderate risk for other exposures (37, 54). In terms of detection bias, 9 studies were appraised as low risk, and 10 as moderate risk, as indicated in supplementary tables S3–S4. Regarding confounding, 4 studies were deemed to have a low risk of bias, having accounted for most confounding variables. As they only adjusted for some confounders in their observed associations, 11 studies were categorized as having a moderate risk of bias. Additionally, 2 studies were considered to have a low risk of bias for some exposures and a moderate risk of bias for some other exposures. Additionally, 1 study was rated to have a moderate risk for one exposure (39) and a high risk for another exposure (54) while another was classified as having a high risk of bias because it failed to adjust for any confounding factors. Concerning attrition bias, 6 studies were considered low risk, 10 moderate risk, and 1 high risk. Moreover, 2 studies were judged to have a low risk of attrition bias for certain exposures, but a moderate risk for others.

### Quantitative synthesis

**Figure 2:**
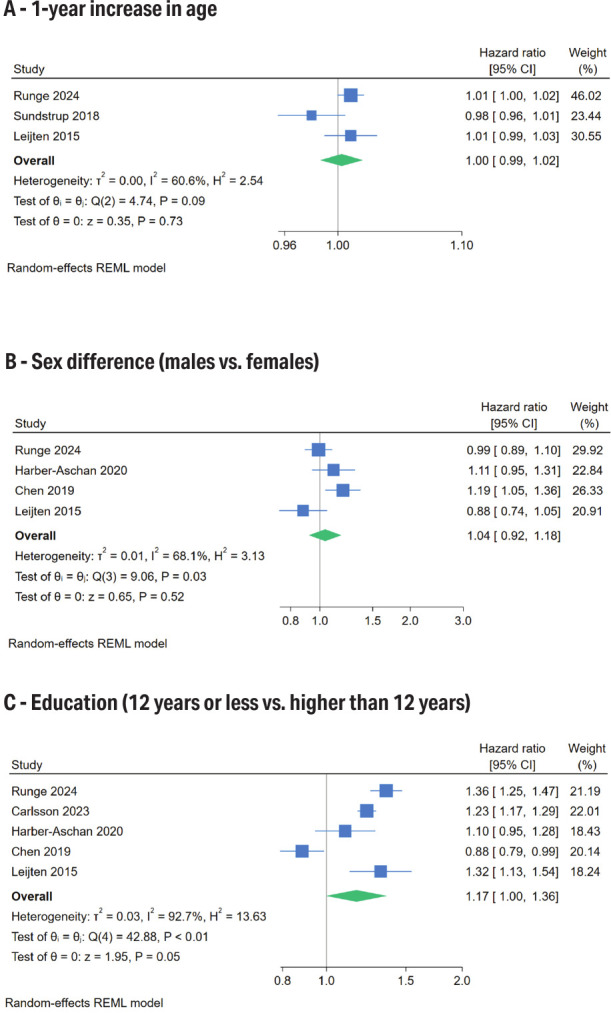
The effects of age (A), sex (B), and education (C) on unemployment risk.

*Sociodemographic factors.* Age and gender were not associated with unemployment risk, as detailed in table 2 and figures 2a and 2b. A meta-analysis of 5 studies, which included a total of 270 759 participants, indicated that individuals with ≤12 years of education had a higher likelihood of being unemployed than those with higher education (HR 1.17, 95% CI 1.00–1.36, figure 2c). Additionally, other research found consistent findings regarding the expected average number of unemployment days (42). Individuals who were unmarried, separated, or widowed faced a greater unemployment risk compared to their married or cohabiting counterparts (HR 1.43, 95% CI 1.14–1.79, supplementary figure S1). The risk was also elevated for immigrants over native-born individuals (HR 1.26, 95% CI 1.11–1.44, supplementary figure S2).

**Table 2:** Risk factors of unemployment. [HR=hazard ratio; CI=confidence interval.]

Risk factor			Studies (N)	Sample	HR	95% CI	I^2^ statistic (%)
Sociodemographic factors							
	Age, 1-year increase		3	68 241	1.00	0.99–1.02	61
	Male sex		4	125 208	1.04	0.92–1.18	68
	Education, ≤12 years vs higher level		5	270 759	1.17	1.00–1.36	93
	Marital status (unmarried, separated, or widowed vs married or cohabiting)		3	66 018	1.43	1.14–1.79	78
	Immigrants vs natives		2	62 043	1.26	1.11–1.44	0
Lifestyle factors							
	Physical activity <1/week vs ≥1/week		2	5509	1.56	1.13–2.15	0
	Smoking						
		Past	2	5509	1.22	0.53–2.80	79
		Current	2	5509	1.61	0.69–3.76	84
	Excessive alcohol intake		2	5509	1.43	0.76–2.66	60
Occupational factors							
	Low job control		3	129 070	1.30	1.17–1.43	26.8
Health conditions							
	Self-rated general health (poor/moderate vs good/excellent)		7	73 421	1.44	1.21–1.70	54
	Mental health conditions		14	175 318	1.48	1.30–1.68	56
		Depressive symptoms	8	46 758	1.42	1.14–1.76	65
	Musculoskeletal disorders		4	116 389	1.05	0.94–1.17	14
	Cardiovascular diseases		4	66 555	1.13	0.96–1.32	0
	Respiratory diseases		4	116 389	1.04	0.95–1.15	0
	Digestive diseases		2	60 787	1.05	0.71–1.53	0
	Diabetes		3	65 969	1.09	0.88–1.35	0
	Neoplasm		2	55 602	1.09	0.92–1.29	0
	Daily activity limitations		6	66 654	1.12	0.99–1.25	0

**Figure 3:**
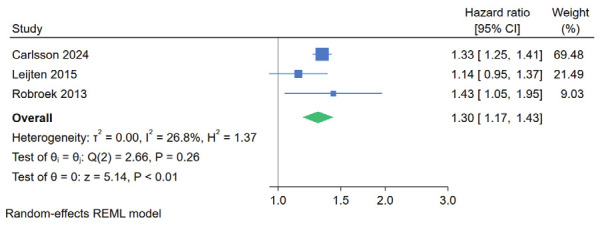
The effect of low job control on unemployment risk.

*Lifestyle and occupational factors.* Workers engaging in physical activity less than once per week were found to be at a higher risk of unemployment than those who exercised more frequently (HR 1.56, 95% CI 1.13–2.15, supplementary figure S3). However, 1 study included in the analysis adjusted the estimate only for age (53). Additionally, workers with low job control were found to be at an increased risk of unemployment (HR 1.30, 95% CI 1.17–1.43, figure 3). Another study corroborated this finding by examining the extent of job control on a continuous scale (46). Smoking and excessive alcohol consumption did not show an association with unemployment risk (table 2).

**Figure 4:**
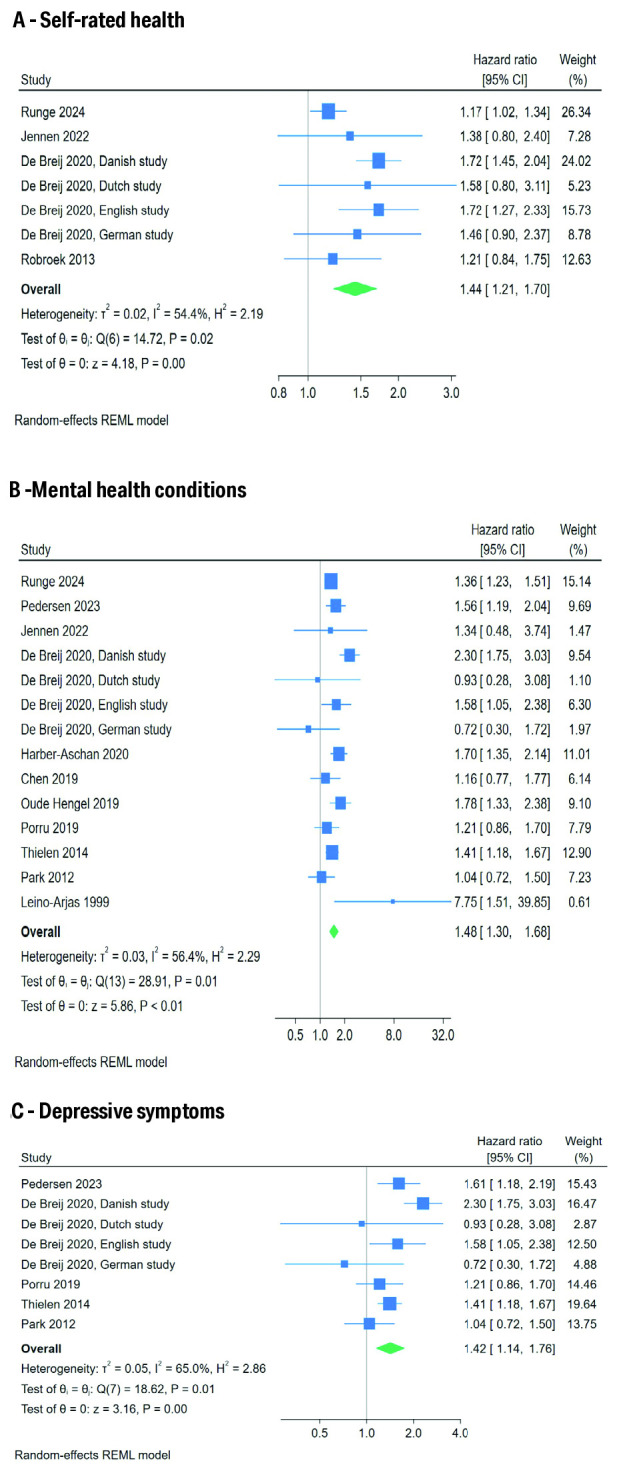
Meta-analysis of studies on the effects of self-rated general health (A), mental health conditions (B) and depressive symptoms (C) on unemployment risk.

*Health conditions.* A meta-analysis of 7 studies, involving a total of 73 421 participants, indicated that employees who rated their overall general health as less than good had a higher risk of unemployment (HR 1.44, 95% CI 1.21–1.70, figure 4A and table 2). Similarly, another study reported that employees who experienced a decrease in self-rated general health within a year at baseline had an increased risk of unemployment during the follow-up period (HR 1.39, 95% CI 1.12–1.73) (20). A meta-analysis involving 14 studies with 175 318 participants found that individuals with mental health conditions had an increased risk of unemployment compared to those without such conditions (HR 1.48, 95% CI 1.30–1.68, figure 4B). Additionally, it was observed that individuals who became unemployed had a yearly reduction in mental health scores by 2.5 points (95% CI -2.9– -2.0) in the period leading up to unemployment (45). A meta-analysis encompassing 8 studies indicated a higher risk of unemployment for workers experiencing depressive symptoms (HR 1.42, 95% CI 1.14–1.76, figure 4C and table 2). Musculoskeletal disorders, cardiovascular and respiratory diseases, digestive system conditions, diabetes, neoplasm, and limitations in daily activities did not show an association with unemployment risk, as detailed in table 2 and supplementary figures S4–S8. Meta-regression showed that the observed differences in effect size across various studies on mental health conditions and depressive symptoms appeared to be independent of the sample sizes and the methodological quality of the studies. However, the observed variation noted in research on self-rated general health was entirely accounted for by adjustments for confounding factors. The combined HR was 1.18 (95% CI 1.05–1.34, I^2^=0%, N=61 694) for the three studies deemed to have a low risk of confounding. In contrast, the HR was 1.69 (95% CI 1.47–1.94, I^2^=0%, N=11 727) across 4 studies categorized as having a moderate risk of confounding. The analysis showed no evidence of publication bias, and employing the trim-and-fill method even suggested the exclusion of two small studies that yielded significant association, absent from the right side of the funnel plots in relation to mental health conditions and depressive symptoms (supplementary figures S9–S13).

### Qualitative synthesis

*Sociodemographic factors.* Identifying an association between occupational class and unemployment risk, 2 studies (13, 17) found that individuals in high-skilled white-collar jobs are less likely to face unemployment compared to other professional groups (13, 17). Furthermore, individuals experiencing financial difficulties had a higher likelihood of becoming unemployed (17). Conversely, a rise in personal income was linked to a lower chance of unemployment (20).

*Lifestyle and occupational factors.* No significant association between excess body mass and self-reported unemployment was found in 1 study. Although obesity (BMI >30 kg/m^2^) was linked to a slightly higher unemployment risk compared to a BMI <25 kg/m^2^, this association was not statistically significant (HR 1.36, 95% CI 0.94–1.99) (11). Another study (53) also reported a non-significant association for obesity (odds ratio [OR] 1.71, 95% CI 0.96–3.02). However, this study compared obese individuals to a control group comprising both normal and overweight subjects. Notably, a significant risk was observed for individuals with a BMI <23 kg/m^2^ (OR 2.35, 95% CI 1.26–4.36) in contrast to those with a BMI of 23–29 kg/m^2^ (53). Additionally, it was found that individuals who became unemployed had, in the years preceding their unemployment, an average annual BMI increase of 0.49 kg/m^2^ (95% CI 0.24–0.73) compared to those who maintained employment (45).

A single study indicated that lifetime exposure to repetitive movements during most work hours, frequent back twisting or bending, and exposure to dust from diverse origins is linked to a higher likelihood of unemployment (19). Conversely, another study revealed no significant association between a physically demanding job or work demanding high time pressure, and the risk of unemployment (11). Workers with effort–reward imbalance (HR 1.49, 95% CI 1.14–1.95) or low job rewards (HR 1.63, 95% CI 1.24–2.15) were at increased risk of unemployment (11). Opposing results regarding the effect of low support from colleagues and/or supervisors on the unemployment risk were found in 2 studies: 1 associated a lack of support with an increased risk of unemployment (21), whereas the other indicated a higher risk of unemployment up to 30 days among individuals with higher social support but found no link with longer unemployment risk (46).

*Health conditions.* A single study investigated the effect of impaired work ability on unemployment risk (51). In contrast to individuals with good/excellent work ability, those with poor work ability were linked to a higher risk of unemployment (OR 4.6, 95% CI 2.2–9.5), whereas moderate work ability (OR 1.1, 95% CI 0.5–2.5) showed no significant association. Nonetheless, this study did not account for any confounding factors that might influence the observed relationship, and the data on unemployment relied on self-reported information. A single study found that climacteric women [incidence rate ratio (IRR) 1.16, 95% CI 1.14–1.18] (36) and women suffering from polycystic ovary syndrome (IRR 1.26, 95% CI 1.23–1.28) (37) experienced a higher number of unemployment days. In contrast, the rate of unemployment days was lower for women with endometriosis (IRR 0.88, 95% CI 0.86–0.91) (38). Workers with metabolic syndrome were at increased risk of unemployment (HR 1.14, 95% CI 1.03–1.25) (13). However, there was no dose–response relationship between the number of metabolic syndrome components and the risk of unemployment (43). Another study found that workers with comorbid chronic conditions were at increased risk of unemployment compared to healthy workers. This risk increased from 24% in individuals with comorbid cardiovascular diseases and musculoskeletal disorders to 57% in workers with comorbid mental and musculoskeletal disorders (20).

## Discussion

The current meta-analytical findings suggest that among individuals aged 40–64 years, suboptimal self-rated general health, mental health conditions, low job control, and insufficient physical activity during leisure time are associated with an increased risk of unemployment. Moreover, the risk of unemployment is higher for individuals with ≤12 years of education, as well as for those who are single, divorced, or widowed, and for immigrants. On the other hand, other lifestyle-related risk factors and treated single physical conditions appear not to contribute to an increased risk of unemployment.

The current meta-analytic findings suggest that suboptimal self-rated general health is a predictor of higher unemployment risk, whereas chronic physical conditions are not. Most studies included in this analysis utilized a common single-item measure for evaluating general health status. It appears that general self-rated health is more likely to be influenced by mental well-being than physical health. Individuals with mental health conditions are more likely to rate their overall health as less than satisfactory compared to those with physical health conditions. A study found that individuals who perceive their mental health as excellent or very good were 93 times more likely to rate their general health similarly, compared to those who consider their mental health to be good or poorer. Conversely, individuals who rate their physical health as excellent or very good were 40 times more likely to report their general health in the same high regard compared to those with a good or poorer assessment of their physical health (55). The included studies did not control for health conditions when examining the association between suboptimal self-rated general health and unemployment. Therefore, the observed association may be attributed to the lack of control for mental health conditions.

There seems to be a reciprocal relationship between mental health and unemployment. A mental health condition can heighten the likelihood of becoming unemployed as suggested by the current meta-analysis, and conversely, being unemployed can increase the risk of developing mental health problems (3, 56). This bidirectional relation highlights the complex interplay between psychological well-being and employment status. In research on unemployment, mental health conditions, especially depressive symptoms, emerged as the most frequently studied risk factors. Also, these conditions were identified as a primary contributor to unemployment among individuals aged 40–64 years.

The current meta-analysis revealed an increased unemployment risk among individuals with low job control, although the impact seems small. Previous studies found that low job control is also associated with an increased risk of disability retirement (57), while high job control is associated with reduced risk of work disability (58). Furthermore, working in low-control jobs is linked to a higher mortality rate (59). Workers in lower occupational classes tend to report lower job control compared to those in higher occupational classes, who conversely report higher job demands (60, 61). The association between low job control and unemployment may be partially explained by a lack of adjustment for socioeconomic status, as the studies included in the meta-analysis did not account for this factor. Furthermore, the review highlights that the influence of potential occupational risk factors on unemployment has not been thoroughly investigated.

An earlier meta-analysis that focused on individuals ≥18 years incorporated 4 studies that assessed the effects of excess body mass and two that examined the role of physical activity in unemployment risk (62). That analysis, consistent with the present study, found no significant link between being overweight or obese and unemployment risk. However, in that systematic review and meta-analysis (62), a qualitative synthesis of 2 studies indicated an increased unemployment risk among individuals with insufficient leisure-time physical activity. Previous studies on the effects of lifestyle risk factors on unemployment risk had some limitations. These studies failed to employ an appropriate comparison group for those at risk, and their chosen comparison groups included at-risk individuals. For example, the obesity comparison group was a mix of overweight and normal weight individuals, which likely led to an underestimation of the link. Conversely, some studies did not account for the most relevant confounding factors, potentially leading to an overestimation of the relationship observed.

### Limitations of the included studies and the review

Most of the studies included in this systematic review enrolled an adequate number of participants, ensuring sufficient statistical power to investigate unemployment risk factors. Each study employed a longitudinal design. Nonetheless, the studies included in this review exhibited certain shortcomings. Many failed to account for most potential confounding factors, with only 32% adjusting their observed associations for most of these factors. These factors include sociodemographic characteristics, lifestyle factors, physical and psychosocial factors at work, and physical and mental health conditions (11, 13, 17, 19, 44). Such oversights could potentially inflate the associations noted. Additionally, previous studies did not consider multiple determinants of unemployment, such as alcohol and substance abuse, work centrality and attitudes, financial demands (eg, having dependent children or debt/wealth). Consequently, we could not study these risk factors. Approximately half of the studies relied on self-reported data for unemployment evaluations, increasing the risk of detection bias. Additionally, there was heterogeneity in both the methods used to measure unemployment and the definitions of unemployment across studies. Given that employment status is dynamic rather than static, it is important to consider the potential risks associated with the duration of unemployment as well. Most studies explored only the incidence of unemployment, with only a few evaluating the risk factors for long-term unemployment. Additionally, some studies did not use a suitable control group for individuals deemed at risk. In certain cases, individuals who were supposed to be part of the at-risk group were included in the control group, which could lead to an underestimation or an absence of the observed association.

The current review also had some limitations. For some risk factors, a limited number of studies was available, resulting in low statistical power. There was a moderate-to-high level of heterogeneity observed within certain meta-analyses. Consequently, to thoroughly investigate some risk factors, additional future large longitudinal studies are needed. Our research was restricted to studies published in English, which may introduce a bias as studies with non-significant findings are more likely to be published in local language journals. Despite this, our analysis encompassed a variety of unemployment risk factors. Most of the studies reviewed found non-significant associations of unemployment with some of these risk factors. In our literature search, we included Google Scholar and two prominent American databases. Due to lack of availability, a European database of Embase or Scopus was not searched. Nevertheless, given that PubMed and Web of Science are leading databases, the likelihood of missing significant studies is minimal. However, this does not preclude the possibility that some studies of lower quality were missed.

### Concluding remarks

The current meta-analytic review underscores the significance of mental health, job autonomy, and recreational physical activity in influencing unemployment risks among individuals in their middle years. Multidimensional interventions are recommended to focus on enhancing mental well-being, increasing job control, and encouraging physical activity during leisure to reduce the risk of unemployment.

## Supplementary material

Supplementary material
