# Supplementary material for: A meta-analysis of unemployment risk factors for middle-aged workers
Source: Scand J Work Environ Health. 2025 Apr 27;51(3):135–45. doi: 10.5271/sjweh.4216 (PMC12068247; doi:10.5271/sjweh.4216)
Supplement: Supplementary material [file SJWEH-51-135-S001.pdf]

A meta-analysis of unemployment risk factors for middle-aged workers[1]

by Rahman Shiri, PhD[2] Joonas Poutanen, MSc, Mikko Härmä, PhD, Jenni Ervasti, PhD, Eija Haukka, PhD

1. Supplementary figures and tables
2. Correspondence to: Rahman Shiri, Finnish Institute of Occupational Health, P.O. Box 18, FI-00032 Työterveyslaitos, Finland. [E-mail: -rahman.shiri@ttl.fi] ORCID ID: 0000-0002-9312-3100

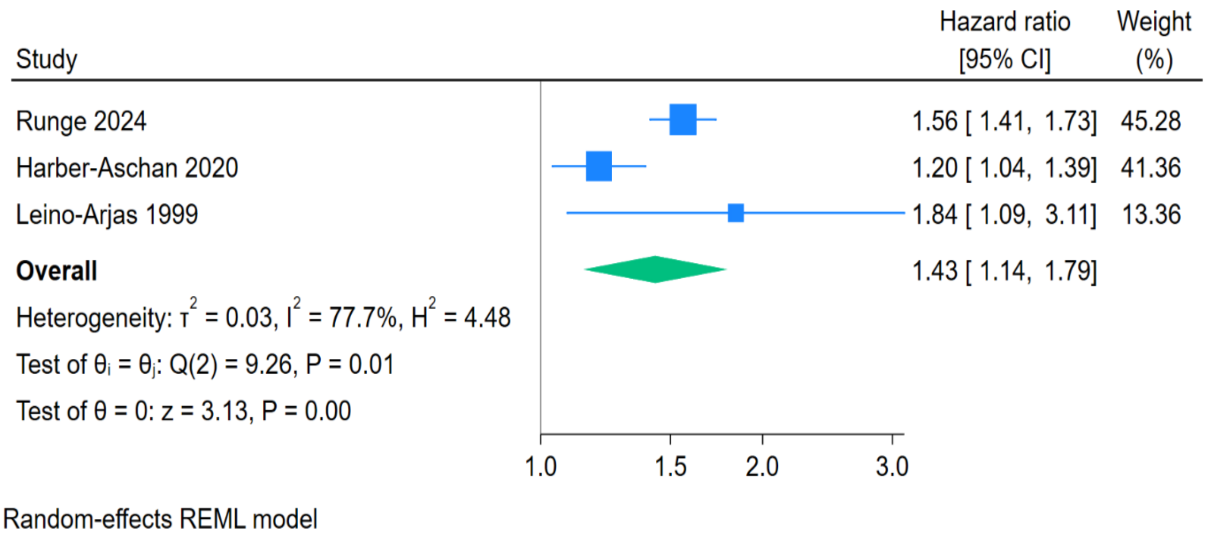

Supplementary Figure S1: The effect of marital status on unemployment risk (unmarried, separated, or widowed vs. married or cohabiting).

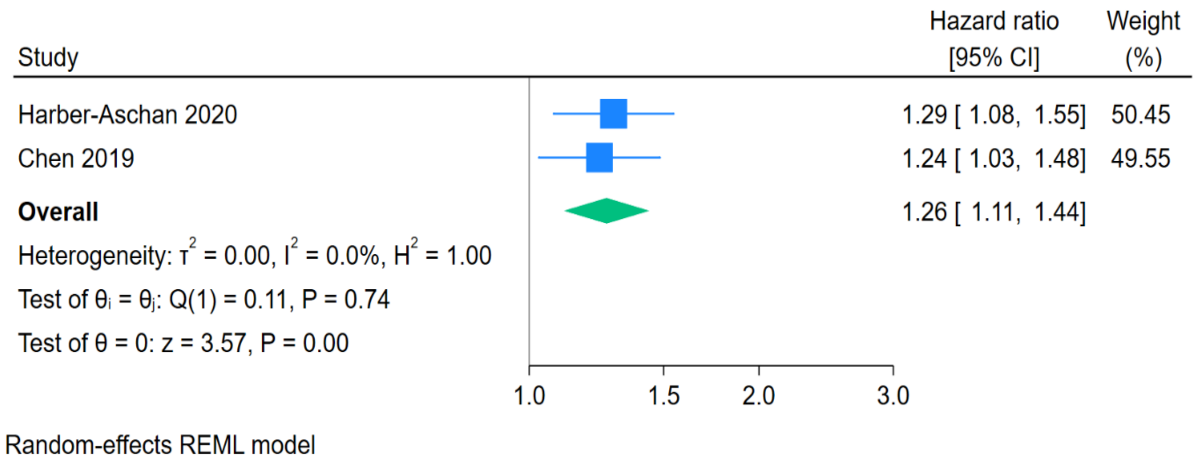

Supplementary Figure S2: The effect of immigration status on unemployment risk (immigrants vs. natives).

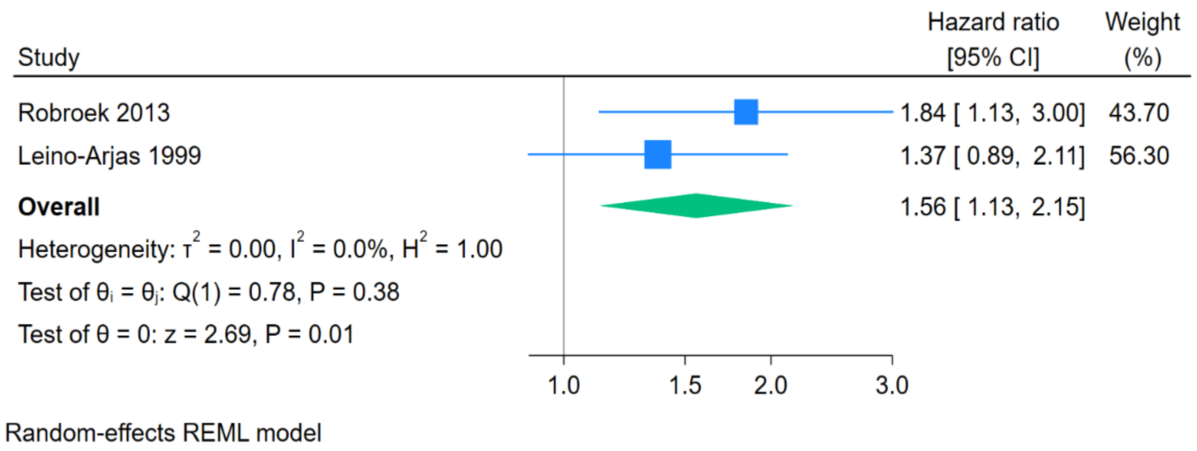

Supplementary Figure S3: The effect of lack of leisure time physical activity on unemployment risk.

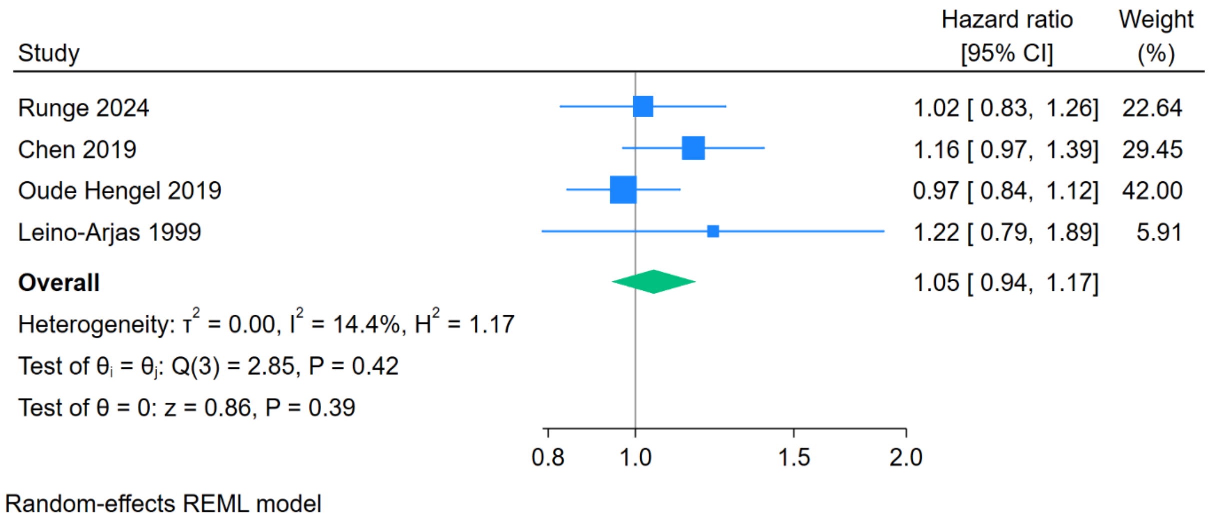

Supplementary Figure S4: The effect of musculoskeletal disorders on unemployment risk.

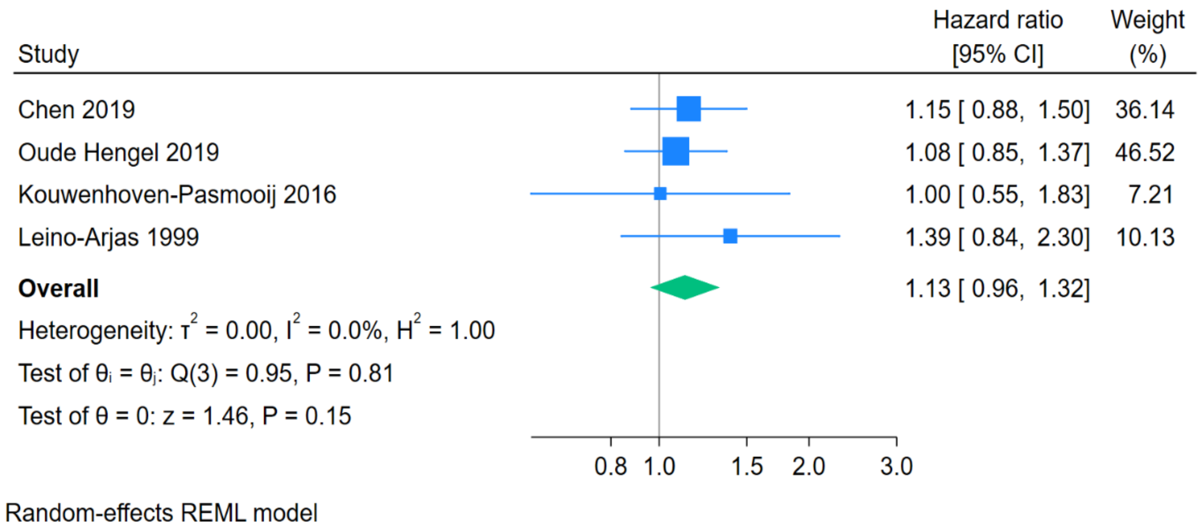

Supplementary Figure S5: The effect of cardiovascular diseases on unemployment risk.

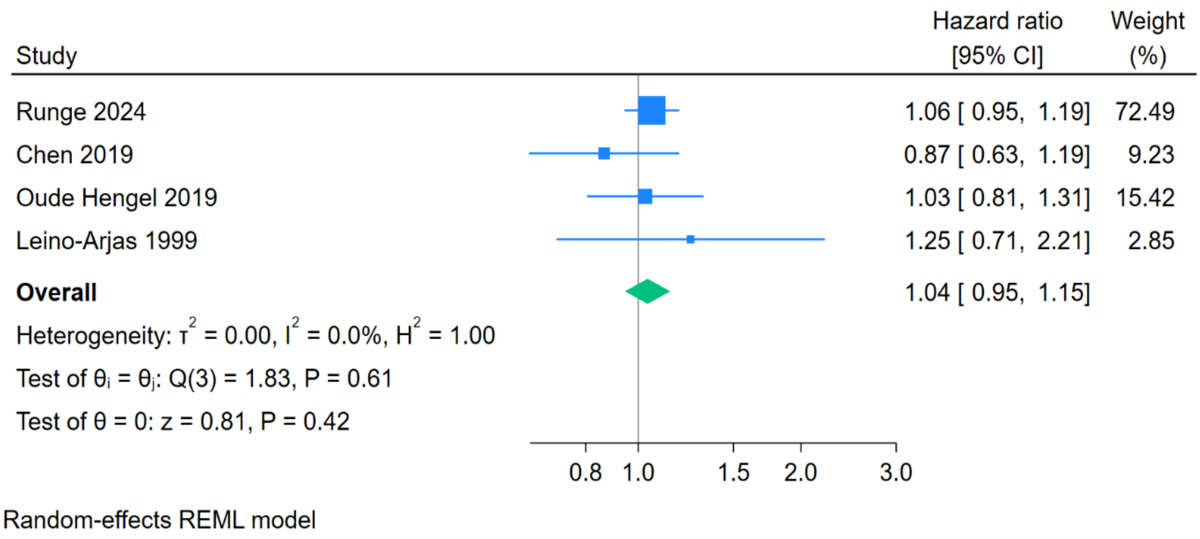

Supplementary Figure S6: The effect of respiratory diseases on unemployment risk.

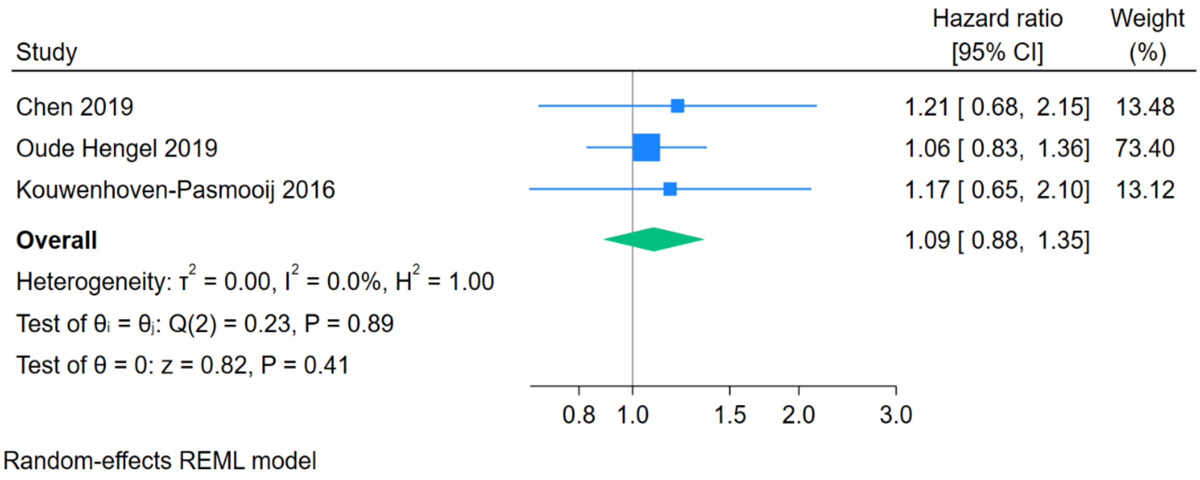

Supplementary Figure S7: The effect of diabetes on unemployment risk.

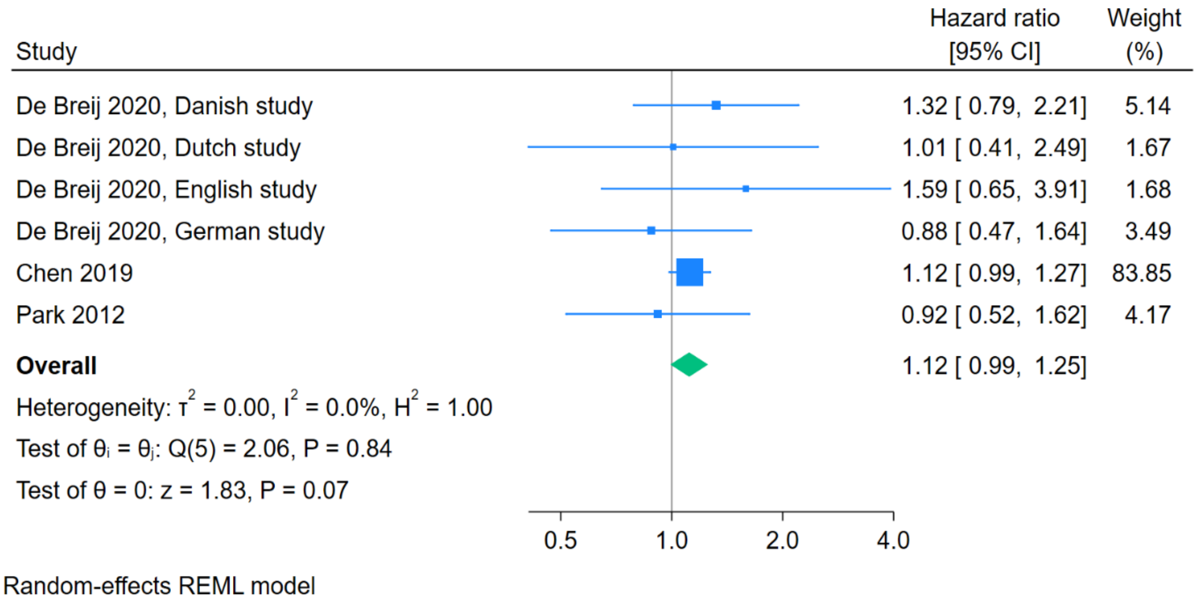

Supplementary Figure S8: The effect of daily activity limitations on unemployment risk.

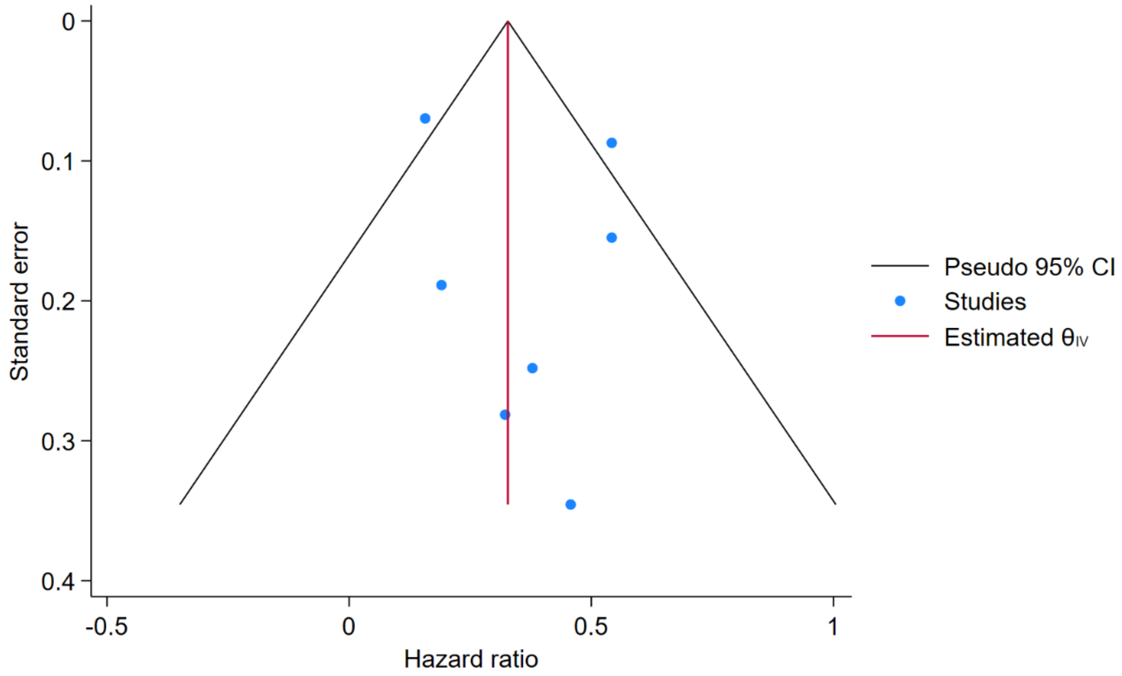

Supplementary Figure S9: Funnel plot for studies examining the association between self-rated general health and unemployment risk.

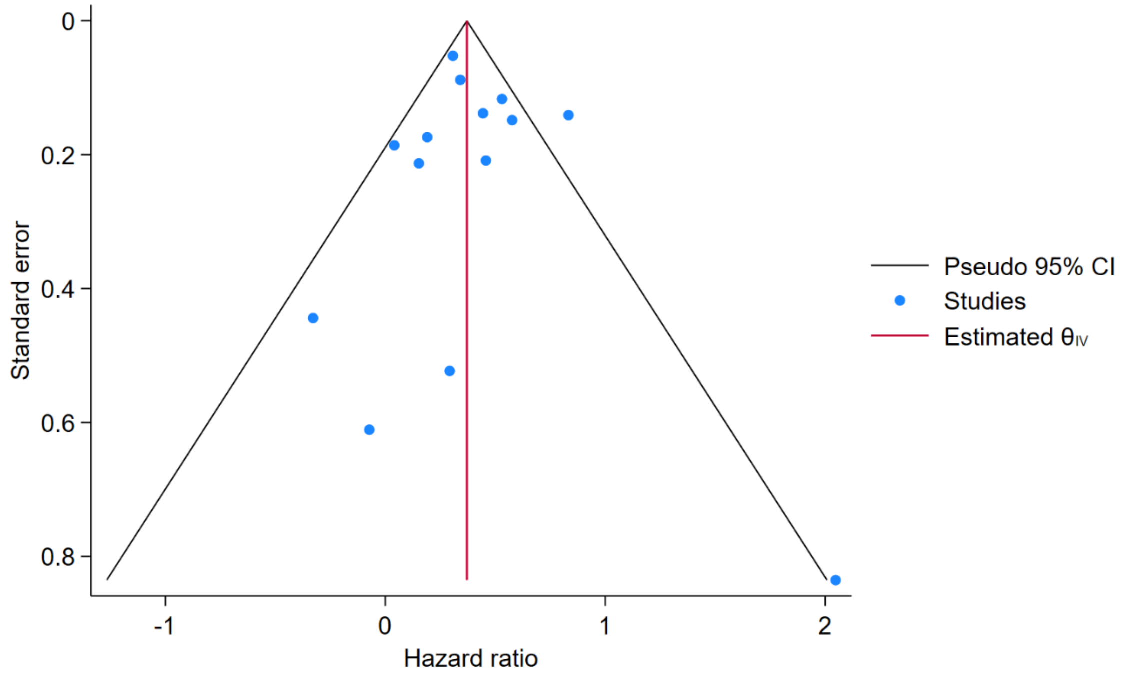

Supplementary Figure S10: Funnel plot for studies examining the association between mental health conditions and unemployment risk.

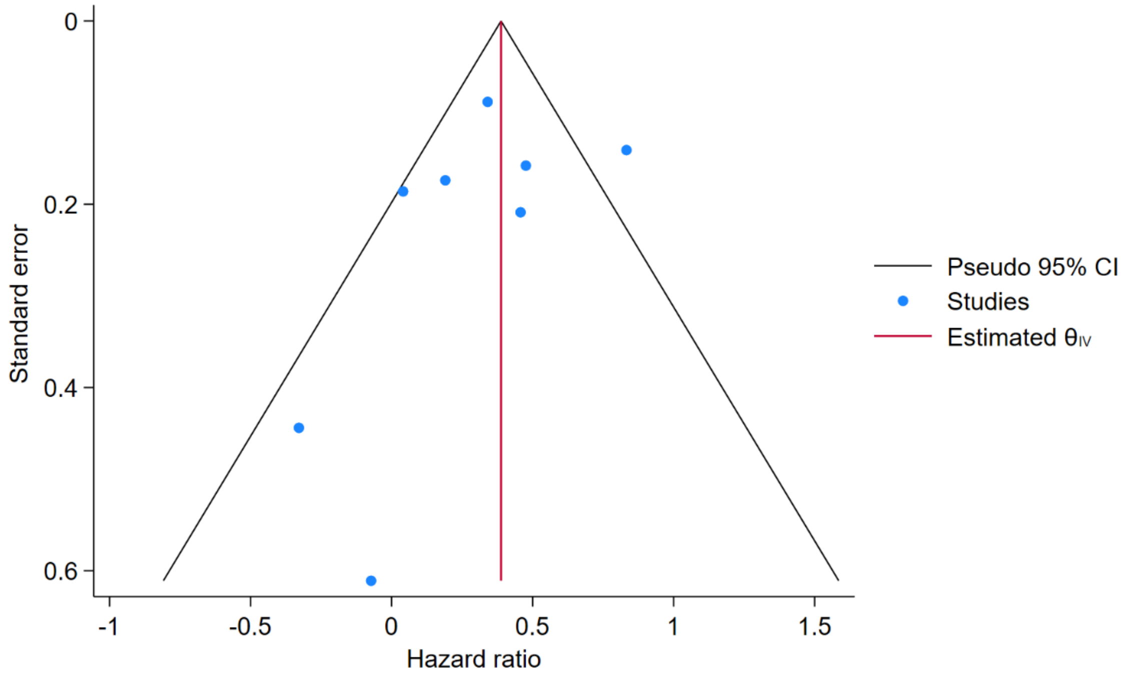

Supplementary Figure S11: Funnel plot for studies examining the association between depressive symptoms and unemployment risk.

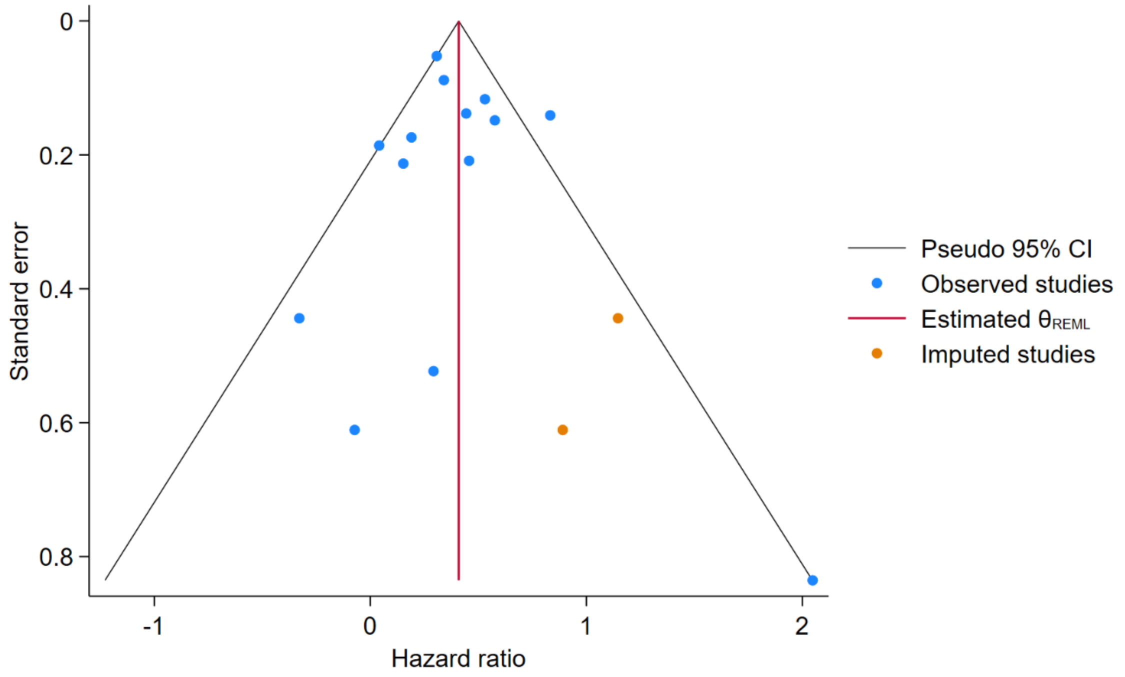

Supplementary Figure S12: Funnel plot of the observed and imputed studies on the association between mental health conditions and unemployment risk.

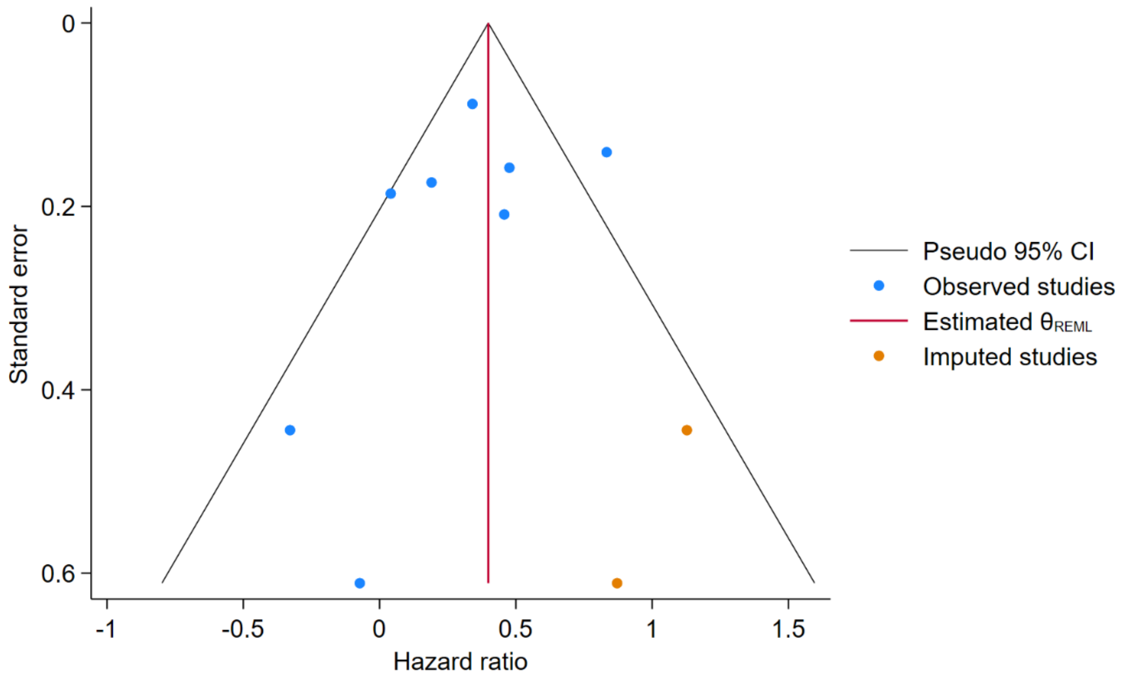

Supplementary Figure S13: Funnel plot of the observed and imputed studies on the association between depressive symptoms and unemployment risk.

Supplementary Table S1: Quality assessment checklist

Type of bias	Criteria definition	Classification (potential for bias)
Selection bias	Sampling method of the study population, representativeness (response rate, difference between responders and non-responders, investigate and control of variables in case of difference between responders and non-responders)	**Low:** Target population defined as representative of the general population or subgroup of the general population (specific age group, women, men, specific geographic area, and specific occupational group) and response rate is 80% or more.<br>**Moderate:** Target population defined as somewhat representative of the general population, a restricted subgroup of the general population, response rate 60%-79%.<br>**High:** Target population defined as "self-referred" or "self-selected"/ volunteers, response rate less than 60%.
Performance bias	Valid and reliable assessment of exposure<br>Assessors blinded to outcome status	**Low:** Exposure was objectively assessed.<br>**Moderate:** A validated questionnaire was employed, or the assessment was reliant on self-reporting.<br>**High**: An appraisal of exposure does not adequately differentiate between exposed and unexposed individuals.
Detection bias	Standard method for outcome assessment<br>The assessor of outcome blinded to exposure status	**Low:** Register-based unemployment.<br>**Moderate:** Self-reported unemployment.
Confounding	Matching two groups<br>Stratification<br>Statistical analysis	**Low:** Controlled for most potential confounding factors, including age and sex.<br>**Moderate:** Controlled for several, but not the majority, of potential confounding factors.<br>**High:** Controlled for only a few confounding factors.
Attrition bias	Withdrawals and drop-out rates<br>Size of missing data	**Low:** Follow up participation rate of 80% or higher or missing data on less than 20%.<br>**Moderate:** Follow up participation rate of 60% –79%, or missing data on 20%–40%.<br>**High:** Follow up participation rate of less than 60%, or missing data on more than 40%.

Supplementary Table S2: The studies included in the review are listed in chronological order based on the most recent report of each study.

No. of studies	First author and year of publication	Country	Follow-up time (years)	Study population	Age range at baseline	Sex	Sample size (in analysis)	Exposure	Unemployment	Results	Adjustment for other covariates
1	Carlsson 2024 (54)	Sweden	14	Individuals born from 1951 to 1953, who completed their compulsory military service in the years 1969–1972 and were alive in 2006.	53–55 years	Men	115 998	Physical workload and job control during 2005 based on job exposure matrices.<br><br>Physical workload and job control were dichotomised using the highest 25% of physical workload and the lowest 25% of job control.	Register based long-term unemployment (≥ 180 days during two consecutive years).	RR 1.40 (1.32–1.48) for high physical workload and 1.33 (1.26–1.42) for low job control.	Unadjusted
1	Carlsson 2023 (39)	Sweden	14	Individuals born from 1951 to 1953, who completed their compulsory military service in the years 1969–1972 and were alive in 2006.	52–54 years	Men.	145 551	Educational level.<br><br>Educational duration was categorized as follows: up to 9 years for primary education, 10 to 11 years for two years of upper secondary school, 12 years for three years of upper secondary school, 13 to 14 years for two years of university education, and 15 years or more for three or additional years of university education.	Registered long-term unemployment (≥ 180 days in one year and ≥ 180 days the following year).	HR 1.31 (95% CI 1.20–1.42) for individuals with 13-14 years of education, 1.42 (95% CI 1.31–1.54) for those with 12 years, 1.59 (95% CI 1.47–1.71) for 10-11 years, and 1.25 (95% CI 1.16–1.36) for those with nine years or less, compared to individuals with 15 or more years of education.<br><br>Estimated HR 1.419 (95% CI 1.356 –1.485) for 12 years or less education compared to individuals with 15 or more years of education.<br><br>Estimated HR 1.2286 (95% CI 1.174 –1.286) for 12 years or less education compared to individuals with higher than 12 years of education.	Childhood factors (parental education, parental occupation, and crowed housing), and late adolescence factors (cognitive ability, stress resilience, BMI ≥ 25 kg/m^2, muscle strength, cardiorespiratory fitness, psychiatric diagnoses, and musculoskeletal diagnoses).
2	Runge 2024 (13) & Runge 2023 (43)	The Netherlands	Median 4.3 (SD 1.9) years.	A representative sample of the general population.	40–64 years.<br><br>Mean age 48.1 ± 5.7 years.	Both.<br><br>54.2% were women.	55 016	Educational level, partner status, perceived general health, metabolic syndrome, diabetes, and chronic conditions.	Self-reported unemployment at follow-up.	HR 1.01 (95% CI 1.00–1.02) for 1-year increase in age, 1.56 (95% CI 1.41–1.73) for not married or partnered vs. married or partnered, and 0.99 (95% CI 0.89–1.09) for male sex.	Age, sex, marital status, education, occupational class, working hours, perceived general health, metabolic

No. of studies	First author and year of publication	Country	Follow-up time (years)	Study population	Age range at baseline	Sex	Sample size (in analysis)	Exposure	Unemployment	Results	Adjustment for other covariates
								Educational attainment was categorized into three levels: low (junior general secondary or preparatory secondary vocational), medium (secondary vocational or senior general secondary or pre-university), and higher (higher vocational or university).<br><br>Perceived general health was evaluated using a five-point scale.<br><br>Metabolic syndrome was defined as having at least three out of five components.<br><br>Self-reported chronic diseases.		HR 1.56 (95% CI 1.39–1.75) for low education and 1.20 (95% CI 1.08–1.34) for medium education compared with high education.<br><br>Estimated HR 1.357 (95% CI 1.254–1.468) for 12 years or less education compared to individuals with higher than 12 years of education.<br><br>HR 1.27 (95% CI 1.15–1.39) for low-skilled white-collar, 0.95 (95% CI 0.82–1.09) for high-skilled blue-collar, and 1.16 (95% CI 1.01–1.34) for low-skilled blue-collar occupations compared with high-skilled white-collar occupations.<br><br>HR 1.02 (95% CI 0.82–1.25) for musculoskeletal disorders, 1.06 (95% CI 0.95–1.19) for pulmonary disease, 1.08 (95% CI 0.91–1.28) for cancer and 1.36 (95% CI 1.23–1.51) for psychiatric disorders.<br><br>HR 1.17 (95% CI 1.02–1.34) for less than good perceived general health and 1.14 (95% CI 1.03–1.25) for metabolic syndrome.<br><br>There was no dose–response relationship between the number of metabolic syndrome components and the risk of unemployment. (43)	syndrome, musculoskeletal disorders, pulmonary disease, cancer, and psychiatric disorders.
3	Saarinen 2024 (36)	Finland	2	A general population	46 years.	Women.	2 661	Climacteric status.	Registered	Unemployment	Education and smoking.

No. of studies	First author and year of publication	Country	Follow-up time (years)	Study population	Age range at baseline	Sex	Sample size (in analysis)	Exposure	Unemployment	Results	Adjustment for other covariates
				sample of people born in 1966 in Northern Finland.				It was based on self-reported last menstruation (>60 days) and blood level of FSH (≥25 IU/L).	unemployment days.	days were higher in climacteric women than preclimacteric women (incidence rate ratio 1.16, 95% CI 1.14-1.18).	
3	Kujanpää 2022 (37)	Finland	2	A general population sample of people born in 1966 in Northern Finland.	46 years.	Women.	1 823	Polycystic ovary syndrome.<br><br>Registered or self-reported physician diagnosed.	Registered unemployment days.	Unemployment days were higher in women with polycystic ovary syndrome than those without the condition (Incidence rate ratio 1.26, 95% CI 1.23–1.28).	Education, marital status, residential area, parity, employment history, BMI, alcohol consumption, smoking, physical activity, and self-rated health.
3	Rossi 2021 (38)	Finland	2	A general population sample of people born in 1966 in Northern Finland.	46 years.	Women.	3 835	Endometriosis.<br><br>Registered or self-reported physician diagnosed.	Registered unemployment days.	The risk of unemployment days was lower in women with endometriosis than those without the condition (incidence rate ratio 0.88, 95% CI 0.86–0.91).	Education, occupational status, marital status, contraceptive use, parity, smoking, alcohol intake, BMI, physical activity, working history and widespread pain.
4	Pedersen 2023 (40)	Denmark	2	A sample of working population.	48–64 years.	Both.<br><br>57.5% were women.	20 683	Depressive and anxiety disorders.<br><br>Depressive disorders were defined as score ≥ 21 on the 12-item Major Depression Inventory.<br><br>Anxiety was measured using the 4-item SCL-ANX4 scale and defined as presence of at least three out of the four	Registered unemployment days.	For employed men, the hazard ratio (HR) for those with only an anxiety disorder was 1.63 (95% CI 0.77–3.44), 1.63 (95% CI 0.96–2.76) for those with only a depressive disorder, and 1.39 (95% CI 0.81–2.37) for those with both depressive and anxiety disorders, in comparison to their counterparts without these conditions.	Education, working time arrangement (part-time vs. full-time), employment sector (private vs. public), body mass index, smoking, history of treatment for a disease, and survey year.

No. of studies	First author and year of publication	Country	Follow-up time (years)	Study population	Age range at baseline	Sex	Sample size (in analysis)	Exposure	Unemployment	Results	Adjustment for other covariates
								dichotomized anxiety symptoms.		For employed women, the HR was 1.28 (95% CI 0.72–2.28) for those with an anxiety disorder only, 1.60 (95% CI 1.09–2.34) for those with a depressive disorder only, and 2.18 (95% CI 1.50–3.15) for those with both conditions, compared to women without these disorders.<br><br>For both sexes combined, the estimated HR was 1.401 (95% CI 0.719–2.211) for those with an anxiety disorder only, 1.610 (95% CI 1.182–2.194) for those with a depressive disorder only, and 1.559 (95% CI 1.189–2.044) for those with a depressive or anxiety disorder only.<br><br>Additionally, men with only an anxiety disorder had 4.8 more days of unemployment (95% CI 2.3–7.3), those with only a depressive disorder had 9.3 more days (95% CI 6.8–11.8), and those with both conditions had 10.2 more days (95% CI 7.7–12.7) than men without these disorders.<br><br>Similarly, women with only an anxiety disorder had 2.3 additional days of unemployment (95% CI 0.5–4.1), those with only a depressive disorder had 7.2 more days (95% CI 5.4–9.0), and those with both conditions had 12.1 more days (95% CI 10.3–13.9) compared to women without these disorders.	

No. of studies	First author and year of publication	Country	Follow-up time (years)	Study population	Age range at baseline	Sex	Sample size (in analysis)	Exposure	Unemployment	Results	Adjustment for other covariates
4	Pedersen 2022 (41)	Denmark	2	A sample of working population.	40–64 years.	Both.<br><br>58.4% were women.	31 719	High physical work demands.<br><br>Work demands were assessed by seven items (score 0 to 100) and grouped into four levels: low (0–10), moderate (11–20), high (21–30), and very high (>30).	Registered unemployment days.	In the age group of 40-49 years, men with moderate work demands experienced an increase of 1.3 days in unemployment (95% CI -0.5 to 3.1), those with high demands saw an increase of 6.9 days (95% CI 5.1–8.7), and those with very high demands had an increase of 3.8 days (95% CI 2.0–5.6) in comparison to their counterparts with low work demands.<br><br>Men between 50-64 years with moderate work demands had an additional 0.7 days of unemployment (95% CI -1.1 to 2.5), those with high demands had 5.2 more days (95% CI 3.4–7.1), and those with very high demands experienced 9.4 additional days (95% CI 7.5–11.2) relative to men with low work demands.<br><br>Women aged 40-49 years with moderate work demands faced 0.8 more days of unemployment (95% CI -1.2 to 2.8), those with high demands also had 0.8 additional days (95% CI -1.2 to 2.8), and those with very high demands encountered 4.0 more days (95% CI 2.0–6.0) compared to those with low work demands.<br><br>Women aged 50-64 years with moderate work demands had 3.7 more days of unemployment (95% CI 1.9–	Education, working time arrangement (part-time vs. full-time), employment sector (private vs. public), body mass index, smoking, physical activity, history of treatment for a disease, depressive symptoms, and survey year.

No. of studies	First author and year of publication	Country	Follow-up time (years)	Study population	Age range at baseline	Sex	Sample size (in analysis)	Exposure	Unemployment	Results	Adjustment for other covariates
										5.5), those with high demands had 5.3 additional days (95% CI 3.5–7.1), and those with very high demands also had 5.3 more days (95% CI 3.5–7.1) in comparison to women with low work demands.	
4	Pedersen 2021 (42)	Denmark	4	A sample of working population.	40–59 years.	Both.<br><br>54.6% were women.	25 293	Level of education.<br><br>Stress perceived from work or personal life.<br><br>Perceived stress was assessed using two items.	Registered unemployment days.	Perceived stress.<br><br>Men aged 40-49 years with work-related stress experienced an increase of 1.9 days (95% CI 1.4–2.5) in unemployment and those with both work and personal life-related stress had an increase of 18.2 days (95% CI 16.8–19.7) in comparison to men without work and personal life-related stress.<br><br>Men aged 50-59 years with work-related stress experienced an increase of 21.0 days (95% CI 19.9–22.0) in unemployment and those with both work and personal life-related stress had an increase of 16.9 days (95% CI 15.5–18.3) in comparison to men without work and personal life-related stress.<br><br>Women aged 40-49 years with work-related stress experienced an increase of 5.7 days (95% CI 5.1–6.3) in unemployment and those with both work and personal life-related stress had an increase of 1.9 days (95% CI 1.5–2.4) in comparison to men without work and personal life-related stress.	Education, working time arrangement (part-time vs. full-time), employment sector (private vs. public), body mass index, smoking, and survey year.

No. of studies	First author and year of publication	Country	Follow-up time (years)	Study population	Age range at baseline	Sex	Sample size (in analysis)	Exposure	Unemployment	Results	Adjustment for other covariates
										Women aged 50-59 years with work-related stress experienced an increase of 10.6 days (95% CI 10.3–10.8) in unemployment and those with both work and personal life-related stress had an increase of 13.1 days (95% CI 12.7–13.4) in comparison to men without work and personal life-related stress.	
										Education	
										In the age group of 40-49, men with a lower education level saw an increase in unemployment by 3.7 days (95% CI 3.2–4.2), whereas their highly educated counterparts experienced 4.6 days less of unemployment (95% CI -4.8, -4.3) compared to those with a medium education level.	
										For men between 50-59 years old, those with a lower education level had 9.7 more days of unemployment (95% CI 9.2–10.2), while the highly educated had 2.8 days less (95% CI -3.1, -2.5) relative to men with a medium education level.	
										Women aged 40-49 with a lower education level encountered 28.7 additional days of unemployment (95% CI 27.2–30.2), and those with a higher education level had 9.7 fewer days (95% CI -9.9, -9.4) compared to women	

No. of studies	First author and year of publication	Country	Follow-up time (years)	Study population	Age range at baseline	Sex	Sample size (in analysis)	Exposure	Unemployment	Results	Adjustment for other covariates
										with a medium education level.<br><br>Women aged 50-59 years with a lower education level experienced an increase of 11.1 days in unemployment (95% CI 10.7–11.4), and those with a higher education level saw a reduction of 8.0 days (95% CI -8.2, -7.8) in comparison to women with a medium education level.	
5	Jennen 2022 (44)	The Netherlands	6	Workers from 45 companies in different sectors (the Maastricht Cohort Study).	45–59.2 years	Both.<br><br>27.6% were women.	1 755	Perceived general health, and chronic conditions.<br><br>Perceived general health was evaluated using a five-point scale.<br><br>Self-reported current chronic physical and mental conditions.	Self-reported unemployment at follow-up.	HR 1.06 (95% CI 0.68–1.66) for chronic physical condition, 1.34 (95% CI 0.48–3.73) for chronic mental condition, and 1.35 (95% CI 0.62–2.93) for comorbid chronic physical and mental conditions.<br><br>HR 1.38 (95% CI 0.80–2.41) for less than good perceived general health.	Age, sex, education, living alone, physically demanding work, psychological job demands, decision latitude, emotional demands, colleagues social support, supervisor social support, working hours, and perceived general health.
6	van de Ven 2022 (45)	The Netherlands	7	A sample of working individuals (the Study on Transitions in Employment, Ability and Motivation [STREAM]).	45–64 years	Both.<br><br>44.7% were women	13 254	BMI, and physical and mental health.<br><br>Physical and mental health were assessed using the 12-item Short-Form Health Survey (SF-12).<br><br>BMI was calculated from self-reported weight and height.	Registered data on transition from paid employment to unemployment.	In the years before to becoming unemployed, individuals who left employment experienced an annual decline in mental health by 2.47 points (95% CI -2.93 to -2.01), a slight decrease in physical health by 0.41 points (95% CI -0.86 to 0.05), and an annual increase in BMI by 0.49 kg/m^2 (95% CI 0.24–0.73) when	Age, sex, and educational level.

No. of studies	First author and year of publication	Country	Follow-up time (years)	Study population	Age range at baseline	Sex	Sample size (in analysis)	Exposure	Unemployment	Results	Adjustment for other covariates
								Physical load and autonomy were assessed using five items, and psychological job demands were assessed using four items.		compared to those who remained employed.<br><br>In the years before the transition from paid employment to unemployment, there were no differences in physical workload, psychological job demands, and autonomy between individuals becoming unemployed and those remaining employed.	
6	Oude Hengel 2019 (16)	The Netherlands	7	A sample of working individuals (STREAM).	45–64 years.<br><br>Mean age 53.8 ± 5.3 years.	Both.<br><br>42.7% were women	9 160	Self-reported current chronic diseases.	Registered unemployment.	HR 1.08 (95% CI 0.85–1.36) for cardiovascular disease, 1.06 (95% CI 0.83–1.36) for diabetes, 1.04 (95% CI 0.49–1.38) for digestive disease, 0.97 (95% CI 0.84–1.12) for musculoskeletal disorder, 1.78 (95% CI 1.33–2.38) for psychological disorder, and 1.03 (95% CI 0.81–1.32) for respiratory disease.<br><br>HR 1.02 (95% CI 0.90–1.15) for a single chronic condition and 1.08 (95% CI 0.92–1.27) for multiple chronic conditions compared with those without chronic illnesses.	Age, sex, educational level, marital status, and other chronic diseases.
6	Leijten 2015 (21)	The Netherlands	3	A sample of working individuals (STREAM).	45–64 years.<br><br>Mean age 53.4 ± 5.1 years.	Both.<br><br>43.4% were women	8 149	Education, physical load, autonomy, and social support.<br><br>Educational attainment was categorized into three levels: low (junior general secondary or preparatory secondary vocational), medium (secondary vocational or senior	Self-reported unemployment at follow-up.	HR 1.01 (95% CI 0.99–1.03) for 1-year increase in age, and 0.88 (95% CI 0.74–1.06) for male sex.<br><br>HR 1.50 (95% CI 1.20–1.88) for low education and 1.17 (95% CI 0.94–1.45) for medium education compared with high level of education.	Age, sex, educational level, marital status, physical load, psychological job demands, lower autonomy, lower support, and chronic diseases

No. of studies	First author and year of publication	Country	Follow-up time (years)	Study population	Age range at baseline	Sex	Sample size (in analysis)	Exposure	Unemployment	Results	Adjustment for other covariates
								general secondary or pre-university), and higher (higher vocational or university).<br><br>Physical load and autonomy were assessed using five items, while psychological job demands and support from colleagues and/or supervisors were assessed using four items. Each of these items was based on a five-point Likert scale.		Estimated HR 1.319 (95% CI 1.129–1.542) for 12 years or less education compared to individuals with higher than 12 years of education.<br><br>HR 0.82 (95% CI 0.68–0.99) for high physical load, 0.87 (95% CI 0.72–1.06) for high psychological job demands, 1.14 (95% CI 0.95–1.38) for low autonomy, and 1.46 (95% CI 1.22–1.75) for low support.	(musculoskeletal, severe headache, circulator, respiratory, digestive, diabetes and psychological).
7	Wang 2022 (46)	Sweden	14 years.<br><br>Mean 8.7 ± 5.4 years.	Twins from the national Swedish Twin Registry, a nearly complete registration of all twins.	46–64 years.	Both.<br><br>51.4% were women.	23 556	Job demands, job control, and social support.<br><br>The assessment of psychosocial working conditions was based on the Swedish psychosocial Job Exposure Matrix and the scores ranged from 1 to 10, which higher scores indicated lower job demands, higher job control and higher social support.	Registered unemployment days: 1–30, 31–365 and more than 365 days.	Unemployment 1-30 days<br><br>HR 0.80 (95% CI 0.68–0.94) for one-unit increase in job demands, 0.91 (95% CI 0.84–0.99) for 1-unit increase in job control, and 1.31 (95% CI 1.07–1.61) for 1-unit increase in social support for age group 46-55 years.<br><br>HR 0.55 (95% CI 0.43–0.71) for one-unit increase in job demands, 1.01 (95% CI 0.87–1.16) for 1-unit increase in job control, and 1.69 (95% CI 1.19–2.40) for 1-unit increase in social support for age group 56-64 years.<br><br>Unemployment 31-365 days<br><br>HR 0.93 (95% CI 0.85–1.01) for one-unit increase in job demands, 0.91 (95% CI 0.87–0.96) for 1-unit increase in job control, and 0.97 (95% CI 0.88–1.07) for 1-unit increase	Sex, education, marital status, children living at home, type of living area, and sickness absence.

No. of studies	First author and year of publication	Country	Follow-up time (years)	Study population	Age range at baseline	Sex	Sample size (in analysis)	Exposure	Unemployment	Results	Adjustment for other covariates
										in social support for age group 46-55 years.<br><br>HR 0.95 (95% CI 0.82–1.09) for one-unit increase in job demands, 0.89 (95% CI 0.83–0.96) for 1-unit increase in job control, and 0.94 (95% CI 0.80–1.10) for 1-unit increase in social support for age group 56-64 years.<br><br>Unemployment >365 days<br><br>HR 0.75 (95% CI 0.69–0.81) for one-unit increase in job demands, 0.88 (95% CI 0.84–0.92) for 1-unit increase in job control, and 0.99 (95% CI 0.90–1.08) for 1-unit increase in social support for age group 46-55 years.<br><br>HR 0.97 (95% CI 0.85–1.11) for one-unit increase in job demands, 0.97 (95% CI 0.90–1.04) for 1-unit increase in job control, and 0.92 (95% CI 0.79–1.07) for 1-unit increase in social support for age group 56-64 years.	
8-11	De Breij 2020 (47)	Danmark, Germany, United Kingdom, and the Netherlands.	Up to 15	A sample of general population.<br><br>Four studies: 1) Longitudinal Aging Study Amsterdam, 2) Danish Longitudinal Study of Ageing), 3) English	50 years or older.	Both	Danmark (N=4 721), Germany (N=1 203), United Kingdom (N=4 508), and the Netherlands (N=1 295).	Perceived general health, functional limitations, and depression.<br><br>Perceived general health was evaluated using a five-point scale.<br><br>To evaluate functional limitations, the studies employed various metrics. The Dutch and Danish studies utilized a set of six	Self-reported unemployment at follow-up.	HR for less than good perceived general health was 1.72 (95% CI 1.45–2.04) for Danmark, 1.46 (95% CI 0.90–2.38) for Germany, 1.72 (95% CI 1.27–2.33) for United Kingdom and 1.58 (95% CI 0.80–3.10) for the Netherlands.<br><br>HRs for functional limitations were 1.32 (95% CI 0.79–2.21) for Danmark, 0.88 (95%	Age, sex, education, partner status, number of working hours, year, and region.

No. of studies	First author and year of publication	Country	Follow-up time (years)	Study population	Age range at baseline	Sex	Sample size (in analysis)	Exposure	Unemployment	Results	Adjustment for other covariates
				Longitudinal Study of ageing, and 4) German Ageing Survey.				items, the English study applied five items, while the German study measured using 10 items from the SF-36 scale.<br><br>For assessing depressive symptoms, the Dutch study incorporated 20 items, the English study 8 items, and the German study 15 items from the Centre for Epidemiologic Studies Depression Scale. In the Danish study, data on depression relied on self-reports of physician-diagnosed depression or instances of depression occurring in the preceding year.		CI 0.47–1.64) for Germany, 1.59 (95% CI 0.65–3.94) for United Kingdom and 1.01 (95% CI 0.41–2.50) for the Netherlands.<br><br>HRs for depression were 2.30 (95% CI 1.75–3.04) for Danmark, 0.72 (95% CI 0.30–1.71) for Germany, 1.58 (95% CI 1.05–2.38) for United Kingdom and 0.93 (95% CI 0.28–3.07) for the Netherlands.	
12	Harber-Aschan 2020 (17)	Sweden	≥ 2	A random sample of general population.	50–62 years.	Both.<br><br>53% were women.	10 416	Sex, educational level, marital status, occupational class, financial strain, limiting long-standing illnesses and common mental disorders.<br><br>Educational attainment was categorized into three levels: basic (completing up to 9th grade), secondary (completing up to 12th grade), and higher education (university).<br><br>Common mental disorders were assessed using the 12-item General Health Questionnaire (score $\geq$4).<br><br>Data on limiting long-standing illness was based	Registered unemployment at follow-up.	HR 1.20 (95% CI 1.04–1.39) for single, divorced or widowed compared with married or in registered partnership.<br><br>HR 1.29 (95% CI 1.08–1.55) for born outside Sweden and 0.90 (95% CI 0.76–1.05) for female sex.<br><br>HR 1.07 (95% CI 0.83–1.37) for primary education and 1.12 (95% CI 0.93–1.36) for secondary education compared with higher education.<br><br>Estimated HR 1.101 (95% CI 0.947–1.282) for primary or secondary education compared with higher education.	Age, sex, education, marital status, country of birth, occupational class, financial strain, employment condition and presence of physical and mental condition.

No. of studies	First author and year of publication	Country	Follow-up time (years)	Study population	Age range at baseline	Sex	Sample size (in analysis)	Exposure	Unemployment	Results	Adjustment for other covariates
								on self-reported health problems limiting the ability to work or do other daily activities.<br><br>Financial strain was defined as borrowing money from family/friends to afford food or rent in the past year.		HR 1.31 (95% CI 1.06–1.62) for intermediate non-manual occupations, 1.86 (95% CI 1.45–2.38) for low non-manual and 1.77 (95% CI 1.37–2.29) for manual compared with high non-manual occupations.<br><br>HR 1.30 (95% CI 1.01–1.69) for financial strain.<br><br>HR 1.00 (95% CI 0.83–1.22) for long-standing illness only, 1.70 (95% CI 1.36–2.15) for a common mental disorder only, and 0.96 (95% CI 0.66–1.41) for comorbid limiting long-standing illnesses and common mental disorder compared with those without both conditions.	
13	Chen 2019 (20)	Canada	5 to 12.<br><br>Mean 5.7 years.	A random sample of general population.	50–62 years.<br><br>Mean age 54.9 years.	Both.<br><br>46.6% were women.	51 627	Sex, education, marital status, immigrant status, income, activity (functional) limitations, a decline in general health within a year, and self-reported physician-diagnosed chronic diseases.<br><br>Educational attainment was categorized into three levels: high school or less (completing up to 12th grade), post-secondary, and university.<br><br>Activity limitations were defined as difficulty in hearing, vision, communication, walking,	Registered nonemployment.<br><br>It was defined as annual earning less than $1000 and receiving no employment or retirement income.	Subdistribution HR 1.193 (standard error [SE] 0.067, P <0.001) for men compared to women.<br><br>HR 1.064 (SE 0.100) for couples and 0.964 (SE 0.166) for lone parents compared to singles.<br><br>HR 0.936 (SE 0.056) for high school or less education and 1.120 (SE 0.100) for university education compared to post-secondary education.<br><br>Estimated HR 0.883 (SE 0.056) for high school or less vs. higher education (post-secondary or university).	Age, sex, marital status, education, immigrant status, self-employed, family size, individual income, other family income, private pension contributions, year of survey, and province.

No. of studies	First author and year of publication	Country	Follow-up time (years)	Study population	Age range at baseline	Sex	Sample size (in analysis)	Exposure	Unemployment	Results	Adjustment for other covariates
								stair climbing, bending, learning, or related actions, caused by a physical or mental problem lasted or is anticipated to last 6 months or longer.		HR 1.236 (SE 0.093, P <0.001) for immigrant compared to Canadian-born.<br><br>HR 0.563 (SE 0.021, P <0.001) for log of individual income, 1.049 (SE 0.016, P <0.001) for log of other family income, and 0.881 (SE 0.01, P <0.001) for log of private pension contributions.<br><br>HR 1.392 (SE 0.111, P <0.001) for drop in general health within a year.<br><br>HR 1.120 (SE 0.065, P <0.01) for activity limitations without health change and 1.734 (SE 0.160, P <0.001) for activity limitations combined with drop in health compared to those with no activity limitations.<br><br>Sex did not influence the relationship between activity limitations and nonemployment. Nonetheless, those with activity limitations and a decline in overall health, but possessing a university degree, had a reduced risk of nonemployment compared to those without activity limitations and only post-secondary education (HR 0.675, SE 0.173, P <0.01).<br><br>Compared to individuals without any chronic condition, HR was 0.865 (SE 0.161) for respiratory diseases	

No. of studies	First author and year of publication	Country	Follow-up time (years)	Study population	Age range at baseline	Sex	Sample size (in analysis)	Exposure	Unemployment	Results	Adjustment for other covariates
										alone. It increased to 1.271 (SE 0.139, P <0.01) when respiratory and musculoskeletal disorders coexisted. For cardiovascular diseases alone, the HR was 1.147 (SE 0.136), which rose to 1.243 (SE 0.113, P <0.001) when combined with musculoskeletal disorders. Mental disorders had an HR of 1.165 (SE 0.213), but when co-occurred with musculoskeletal disorders, it increased to 1.570 (SE 0.153, P <0.001). Diabetes alone presented an HR of 1.207 (SE 0.294), and in conjunction with cardiovascular diseases, had an HR of 1.334 (SE 0.174, P <0.01). Musculoskeletal disorders alone had an HR of 1.160 (SE 0.092, P <0.05), but when associated with other conditions (excluding respiratory, cardiovascular, diabetes, mental, and digestive diseases), it increased to 1.424 (SE 0.139, P <0.001). Digestive diseases alone showed an HR of 1.051 (SE 0.285), which slightly increased to 1.170 (SE 0.133) when paired with musculoskeletal disorders.	
14	Porru 2019 (48)	11 European countries (SHARE study): Austria, Belgium, Denmark, France, Germany,	4	A random sample of general population.<br><br>The sampling designs varied from simple random selection	50–64 years.<br><br>Mean age 55.0 years.	Both.<br><br>41.8% were women	5 263	Depressive symptoms.<br><br>The evaluation of depressive symptoms was conducted through the 12-item EURO-D scale, and a score of four or more	Self-reported unemployment at follow-up.	Hazard ratio 1.21 (95% CI 0.86-1.70) for total sample, 1.37 (95% CI 0.81-2.31) for men and 1.10 (95% CI 0.70-1.72) for women.	Age, sex, education, marital status, time pressure, job control, and job rewards.

No. of studies	First author and year of publication	Country	Follow-up time (years)	Study population	Age range at baseline	Sex	Sample size (in analysis)	Exposure	Unemployment	Results	Adjustment for other covariates
		Greece, Italy, Spain, Sweden, Switzerland, and the Netherlands		of households to multistage sampling designs.				indicated a potential risk for depression.			
14	Reeuwijk 2017 (49)	11 European countries (SHARE study)	6	A random sample of general population.	50–64 years.<br><br>Mean age 55.3 ± 3.6 years.	Both.<br><br>43.8% were women.	5 273	Perceived general health, assessed by using a 5-point scale.	Self-reported unemployment at follow-up.	Cause-specific Cox hazard ratio was 1.43 (95% CI 1.04–1.97) for moderate or poor heath compared with good or excellent general health.<br><br>Fine & Gray subdistribution hazard ratio was 1.32 (95% CI 0.96–1.83) for moderate or poor health.	Age, sex, education, marital status, and welfare state regime.
14	Kouwenhoven-Pasmooij 2016 (50)	11 European countries (SHARE study)	6	A random sample of general population.	50–64 years.<br><br>Mean age 55.4 ± 3.6 years.	Both.<br><br>44.9% were women.	5 182	Self-reported stroke, heart disease and diabetes diagnosed by a physician.	Self-reported unemployment at follow-up.	OR 1.17 (95% CI 0.65–2.09) for diabetes, 0.91 (95% CI 0.46–1.82) for heart disease and 1.36 (95% CI 0.41–4.53) for stroke.<br><br>The estimated OR 1.005 (95% CI 0.553–1.825) for cardiovascular disease (heart disease or stroke).	Age, sex, education, marital status, and European region.
14	Robroek 2013 (11)	11 European countries (SHARE study)	4	A random sample of general population.	50–64 years.<br><br>Mean age 55.2 ± 3.5 years.	Both.<br><br>44% were women	4 923	Perceived general health, assessed by using a 5-point scale.<br><br>BMI was based on self-reported height and weight and grouped into normal (<25 kg/m^2), overweight, (≥25–<30 kg/m^2) and obese (≥30 kg/m^2).<br><br>Smoking was grouped into never, past, and current.<br><br>Lack of moderate or vigorous physical activity was based on self-reported	Self-reported unemployment at follow-up.	HR 1.21 (95% CI 0.84–1.76) for less than good perceived general health, 1.07 (95% CI 0.79–1.46) for overweight, 1.36 (95% CI 0.94–1.99) for obesity, 1.84 (95% CI 1.13–3.01) for lack of physical activity, 1.10 (95% CI 0.74–1.64) for excessive alcohol use, 1.43 (95% CI 1.05–1.96) for low job control, 1.63 (95% CI 1.24–2.15) for low job rewards, 0.84 (95% CI 0.60–1.17) for past smoking, 1.08 (95% CI 0.78–1.49) for current smoking, 0.86 (95% CI 0.65–1.13) for physically	Age, sex, education, marital status, perceived general health, BMI, lack of physical activity, excessive alcohol intake, low job control and low job rewards.

No. of studies	First author and year of publication	Country	Follow-up time (years)	Study population	Age range at baseline	Sex	Sample size (in analysis)	Exposure	Unemployment	Results	Adjustment for other covariates
								activity less than once a week.<br><br>Excessive alcohol use was defined as intake of >2 glasses of alcohol beverages ≥5 days a week in the past 6 months.<br><br>Psychosocial factors at work were assessed by some items of the Job Content Questionnaire. Job control was assessed by two items and job rewards by five items. Physically demanding job and time pressure were assessed by a single 4-point scale.		demanding job, 0.82 (95% CI 0.63–1.08) for high time pressure, 1.32 (95% CI 0.89–1.96) for low job control in combination with high demands, and 1.49 (95% CI 1.14–1.95) for effort–reward imbalance.	
15	Sundstrup 2018 (19)	Denmark	4 to 6	A sample of middle-aged people living in the eastern parts of the country.	49–63 years.<br><br>Mean age 54.3 ± 3.8 years.	Both.<br><br>30% were women.	5 076	Age and self-reported lifetime physical workload factors.<br><br>Physical work demands over a lifetime were categorized into four levels: low, moderate, high, and very high.<br><br>Specific workload factors included: 1) lifting or moving heavy objects or individuals, 2) performing repetitive tasks for the majority of work hours, 3) operating tools that produce hand vibrations,4) exerting force to pull or push substantial weights, 5) frequent twisting or bending of the back, 6) being in environments with dust from various sources such as cement, demolition	Registered unemployment.	HR 0.98 (95% CI 0.95–1.00) for 1-year increase in age.<br><br>HR 1.19 (95% CI 0.99–1.42) for moderate physical demands, 1.15 (95% CI 0.95–1.40) for high demands and 1.23 (95% CI 0.96–1.57) for very high demands compared to low work demands (sedentary work).<br><br>HR 1.52 (95% CI 1.20–1.93) for short-time (<10 years) and HR 1.22 (95% CI 1.00–1.49) for long-time ≥20 years) exposures to repetitive movements.<br><br>HR 1.53 (95% CI 1.20–1.95) for short-time and 1.40 (95% CI 1.09–1.79) for long-time exposures to dust.	Age, sex, socioeconomic position, psychosocial work environment (quantitative demands, emotional demands, decision authority, work pace, role conflicts, rewards, and possibilities for development), lifestyle factors (physical activity, BMI, and smoking), chronic diseases (back disease, cancer, and chronic

No. of studies	First author and year of publication	Country	Follow-up time (years)	Study population	Age range at baseline	Sex	Sample size (in analysis)	Exposure	Unemployment	Results	Adjustment for other covariates
								sites, mineral fibers, wood, and biological materials, 7) welding smoke, and 8) diesel fumes.		HR 1.56 (95% CI 1.24–1.95) for frequent twisting or bending of the back for less than 10 years.<br><br>Other workload factors were not significantly associated with unemployment.	depression or anxiety), previous long-term sick leave.
16	Thielen 2014 (15)	Denmark	2	A random sample of general population.	40–50 years.	Both.<br><br>49.8% were women.	5 785	Physical work demands, and depressive symptoms.<br><br>Physical work demands were assessed using four items.<br><br>Depressive symptoms were assessed using the 10-item Major Depression Inventory.	Registered unemployment.	Odds ratio 1.45 (95% CI 1.08–1.95) for moderate and 2.17 (95% CI 1.57–2.98) for high work demands compared to low demands.<br><br>OR 1.02 (95% CI 0.80–1.28) for depressive symptoms score 3-8, 2.08 (95% CI 1.54–2.80) for depressive symptoms score 9-19, and 1.99 (95% CI 1.22–3.26) for depressive symptoms score >19 compared to depressive symptoms score <3.<br><br>Estimated OR 1.406 (95% CI 1-183–1.672) for depressive symptoms score 3 or higher.<br><br>OR 1.63 (95% CI 1.27–2.08) for physical demands only, 1.93 (95% CI 1.40–2.66) for depressive symptoms (score >8) only, and 3.09 (95% CI 2.21–4.31) for joint physical demands and depressive symptoms compared to low demands and depressive symptoms score <9).	Age, sex, marital status, vocational education, alcohol consumption, smoking, obesity, back pain and depressive symptoms.
17	Bethge 2012 (51)	Germany	1	A random sample of white-collar workers who were registered in the German	45–59 years.<br><br>Mean age 50.9 ± 4.1 years.	Both.<br><br>44.1% were women.	1 036	Work ability.<br><br>It was assessed by the 7-item work ability index.	Self-reported unemployment at follow-up.	OR was 1.1 (95% CI 0.5–2.5) for moderate and 4.6 (95% CI 2.2–9.5) for poor work ability compared with good/excellent work ability.	Unadjusted

No. of studies	First author and year of publication	Country	Follow-up time (years)	Study population	Age range at baseline	Sex	Sample size (in analysis)	Exposure	Unemployment	Results	Adjustment for other covariates
				Pension Insurance Fund.							
18	Park 2012 (52)	South Korea	2	A random sample of general population.	45 years or older.	Both.<br><br>35.9% were women.	3 300	Perceived general health, assessed by using a 5-point scale.<br><br>Depression was defined as a score >4 on the 10-item Center for Epidemiological Studies Depression Scale.<br><br>The instrumental activities of daily living included seven items for common everyday activities.	Self-reported unemployment at follow-up.	OR 1.18 (95% CI 0.60–2.35) for men and 0.77 (95% CI 0.46–1.30) for women for poor perceived general health compared with moderate to excellent health.<br><br>OR 1.09 (95% CI 0.62–1.93) for men and 1.01 (95% CI 0.63–1.63) for women for depression.<br><br>Estimated OR 1.042 (95% CI 0.724–1.501) for both sexes combined.<br><br>OR 0.92 (95% CI 0.46–1.85) for men and 1.61 (95% CI 0.86–2.99) for women for traffic accident injury.<br><br>OR 0.88 (95% CI 0.42–1.80) for men and 0.98 (95% CI 0.39–2.46) for instrumental activities of daily living disability.<br><br>For daily activity limitations, the estimated OR 0.917 (95% CI 0.518–1.623) for both sexes combined.	Age, marital status, education, type of health insurance, household income, and type of employment contract.
19	Leino-Arjas 1999 (53)	Finland	4	Male blue-collar workers from 11 construction companies.	40–59 years	Men	586	High alcohol consumption was defined as the upper 10% of the distribution of alcohol intake.<br><br>BMI was based on self-reported weight and height.	Self-reported unemployment lasting longer than 24 months during the follow-up.	Multivariable ORs:<br><br>OR 1.12 (95% CI 0.62–2.03) for workers aged 45-49 years, 2.00 ((95% CI 1.08–3.71) for workers aged 50-54 years, and 1.39 ((95% CI 0.55–3.52) for workers aged 55-59 years compared with those aged 40-44 years.	Age, marital status, previous unemployment, spending three years or less in the current job, BMI, smoking, high alcohol consumption, frequent stress

No. of studies	First author and year of publication	Country	Follow-up time (years)	Study population	Age range at baseline	Sex	Sample size (in analysis)	Exposure	Unemployment	Results	Adjustment for other covariates
								Self-reported currently having a disease diagnosed by a physician.<br><br>Infrequent physical exercise was defined as less than once a week for at least half an hour.		OR 1.84 (95% CI 1.09–3.12) for unmarried, separated, or widowed compared with married or cohabiting.<br><br>OR 3.09 (95% CI 1.88–5.08) for spending three years or less in the current job.<br><br>OR 1.98 (95% CI 0.99–3.97) for past smoking and 2.58 (95% CI 1.42–4.69) for current smoking.<br><br>OR 2.11 (95% CI 1.05–4.25) for high alcohol intake.<br><br>OR 2.35 (95% CI 1.26–4.36) for BMI <23 kg/m^2 and 1.71 (95% CI 0.96–3.02) for BMI >29 kg/m^2 compared with BMI 23-29 kg/m^2.<br><br>OR 7.75 (95% CI 1.51–39.93) for mental disorder.<br><br>OR 1.99 (95% CI 1.01–3.93) for skin disease.<br><br>Age-adjusted ORs:<br><br>OR 1.37 (95% CI 0.89–2.11) for infrequent physical exercise.<br><br>OR 1.39 (95% CI 0.84–2.30) for cardiovascular disease, 1.22 (95% CI 0.79–1.89) for musculoskeletal disorder, 1.25 (95% CI 0.71–2.21) for respiratory disease, and 1.42 (95% CI 0.45–4.53) for neoplasms.	symptoms, mental disorder, and skin disease.

Supplementary Table S3: The quality assessment (risk of bias)

No. of studies	First author, year (reference no.)	Selection	Performance	Detection	Confounding	Attrition
1	Carlsson 2024 (54)	Low risk	Moderate risk	Low risk	High risk	Low risk
1	Carlsson 2023 (39)	Low risk	Low risk	Low risk	Moderate risk	Low risk
2	Runge 2024 (13) & Runge 2023 (43)	Moderate risk	Moderate risk	Moderate risk	Low risk	Moderate risk
3	Saarinen 2024 (36)	High risk	Low risk	Low risk	Moderate risk	Low risk
3	Kujanpää 2022 (37)	Moderate risk	Moderate risk	Low risk	Low risk	Moderate risk
3	Rossi 2021 (38)	Moderate risk	Low risk	Low risk	Low risk	Low risk
4	Pedersen 2023 (40), Pedersen 2022 (41) & Pedersen 2021 (42)	High risk	Moderate risk	Low risk	Moderate risk	Moderate risk
5	Jennen 2022 (44)	High risk	Moderate risk	Moderate risk	Low risk	Low risk
6	van de Ven 2022 (45)& Oude Hengel 2019 (16)	Moderate risk	Moderate risk	Low risk	Moderate risk	Low risk
6	Leijten 2015 (21)	Moderate risk	Moderate risk	Moderate risk	Moderate risk	Moderate risk
7	Wang 2022 (46)	Moderate risk	Moderate risk	Low risk	Moderate risk	Low risk
8-11	De Breij 2020 (47), the Danish study	Moderate risk	Moderate risk	Moderate risk	Moderate risk	Moderate risk
8-11	De Breij 2020 (47), the Dutch study	Moderate risk	Moderate risk	Moderate risk	Moderate risk	Moderate risk
8-11	De Breij 2020 (47), the English study	Moderate risk	Moderate risk	Moderate risk	Moderate risk	Moderate risk
8-11	De Breij 2020 (47), the German study	High risk	Moderate risk	Moderate risk	Moderate risk	High risk
12	Harber-Aschan 2020 (17)	Moderate risk	Moderate risk	Low risk	Low risk	Moderate risk
13	Chen 2019 (20)	Moderate risk	Moderate risk	Low risk	Moderate risk	Moderate risk
14	Porru 2019 (48), Reeuwijk 2017 (49) & Kouwenhoven-Pasmooij 2016 (50)	Moderate risk	Moderate risk	Moderate risk	Moderate risk	Moderate risk
14	Robroek 2013 (11)	Moderate risk	Moderate risk	Moderate risk	Low risk	Moderate risk
15	Sundstrup 2018 (19)	High risk	Moderate risk	Low risk	Low risk	Low risk
16	Thielen 2014 (15)	Moderate risk	Moderate risk	Low risk	Moderate risk	Low risk
17	Bethge 2012 (51)	High risk	Low risk	Moderate risk	High risk	Moderate risk
18	Park 2012 (52)	Moderate risk	Moderate risk	Moderate risk	Moderate risk	Low risk
19	Leino-Arjas 1999 (53)	Low risk	Moderate risk	Moderate risk	Moderate risk	Moderate risk

Low risk (green)
Moderate risk (yellow)
High risk (red)

Supplementary Table S4: The risk of performance bias.

No. of studies	First author, year (reference no.)	Overall	BMI	Smoking, physical activity	Occupational factors	Work ability, perceived health, and perceived stress	Metabolic syndrome	Medical conditions
1	Carlsson 2024 (54)	🟡			🟡			
1	Carlsson 2023 (39)	🟢						
2	Runge 2024 (13) & Runge 2023 (43)	🟡			🟡	🟡	🟢	🟡
3	Saarinen 2024 (36)	🟢						🟢
3	Kujanpää 2022 (37)	🟡						🟡
3	Rossi 2021 (38)	🟢						🟢
4	Pedersen 2023 (40), Pedersen 2022 (41) & Pedersen 2021 (42)	🟡			🟡	🟡		🟡
5	Jennen 2022 (44)	🟡				🟡		🟡
6	van de Ven 2022 (45) & Oude Hengel 2019 (16)	🟡	🟡		🟡			🟡
6	Leijten 2015 (21)	🟡			🟡			🟡
7	Wang 2022 (46)	🟡			🟡			
8-11	De Breij 2020 (47), the Danish study	🟡				🟡		🔴
8-11	De Breij 2020 (47), the Dutch study	🟡				🟡		🟡
8-11	De Breij 2020 (47), the English study	🟡				🟡		🟡
8-11	De Breij 2020 (47), the German study	🟡				🟡		🟡
12	Harber-Aschan 2020 (17)	🟡						🟡
13	Chen 2019 (20)	🟡				🟡		🟡
14	Porru 2019 (48), Reeuwijk 2017 (49) & Kouwenhoven-Pasmooij 2016 (50)	🟡				🟡		🟡
14	Robroek 2013 (11)	🟡	🟡	🟡	🟡	🟡		
15	Sundstrup 2018 (19)	🟡			🟡			
16	Thielen 2014 (15)	🟡			🟡			🟡
17	Bethge 2012 (51)	🟢				🟢		
18	Park 2012 (52)	🟡				🟡		🟡
19	Leino-Arjas 1999 (53)	🟡	🟡	🟡				🟡

Low risk 🟢
Moderate risk 🟡
High risk 🔴

References

1. International Labour Organization. World Employment and Social Outlook: Trends 2024. Available from: https://www.ilo.org/publications/flagship-reports/world-employment-and-social-outlook-trends-2024.

2. Eurostat. Unemployment by sex and age – annual data 2023. Available from: https://ec.europa.eu/eurostat/databrowser/view/une_rt_a/default/table?lang=en&category=labour.employ.lfsi.une.

3. Kim TJ, von dem Knesebeck O. Perceived job insecurity, unemployment and depressive symptoms: a systematic review and meta-analysis of prospective observational studies. Int Arch Occup Environ Health 2016 May;89(4):561–73. https://doi.org/10.1007/s00420-015-1107-1.

4. Picchio M, Ubaldi M. Unemployment and health: A meta-analysis. J Econ Surv 2024;38:1437–72. https://doi.org/10.1111/joes.12588.

5. Gedikli C, Miragliab M, Connolly S, Bryand M, Watson D. The relationship between unemployment and wellbeing: an updated meta-analysis of longitudinal evidence. Eur J Work Organ Psychol 2023;32(1):128–44. https://doi.org/10.1080/1359432X.2022.2106855.

6. Tøge AG. Health effects of unemployment in Europe (2008-2011): a longitudinal analysis of income and financial strain as mediating factors. Int J Equity Health 2016 May;15:75. https://doi.org/10.1186/s12939-016-0360-6.

7. OECD. (2024a), Society at a Glance 2024: OECD Social Indicators, OECD Publishing, Paris, https://doi.org/10.1787/918d8db3-en.

8. OECD. (2024b), Promoting Better Career Choices for Longer Working Lives: Stepping Up Not Stepping Out, Ageing and Employment Policies, OECD Publishing, Paris, https://doi.org/10.1787/1ef9a0d0-en.

9. Silverstein M. Meeting the challenges of an aging workforce. Am J Ind Med 2008 Apr;51(4):269–80. https://doi.org/10.1002/ajim.20569.

10. Nygaard NB, Thomsen GF, Rasmussen J, Skadhauge LR, Gram B. Workability in the Ageing Workforce-A Population-Based Cross-Sectional Study. Int J Environ Res Public Health 2021 Nov;18(23):12656. https://doi.org/10.3390/ijerph182312656.

11. Robroek SJ, Schuring M, Croezen S, Stattin M, Burdorf A. Poor health, unhealthy behaviors, and unfavorable work characteristics influence pathways of exit from paid employment among older workers in Europe: a four year follow-up study. Scand J Work Environ Health 2013 Mar;39(2):125–33. https://doi.org/10.5271/sjweh.3319.

12. van Rijn RM, Robroek SJ, Brouwer S, Burdorf A. Influence of poor health on exit from paid employment: a systematic review. Occup Environ Med 2014 Apr;71(4):295–301. https://doi.org/10.1136/oemed-2013-101591.

13. Runge K, van Zon SK, Henkens K, Bültmann U. Metabolic syndrome and poor self-rated health as risk factors for premature employment exit: a longitudinal study among 55 016 middle-aged and older workers from the Lifelines Cohort Study and Biobank. Eur J Public Health 2024 Apr;34(2):309–15. https://doi.org/10.1093/eurpub/ckad219.

14. Kaspersen SL, Pape K, Vie GA, Ose SO, Krokstad S, Gunnell D et al. Health and unemployment: 14 years of follow-up on job loss in the Norwegian HUNT Study. Eur J Public Health 2016 Apr;26(2):312–7. https://doi.org/10.1093/eurpub/ckv224.

15. Thielen K, Nygaard E, Andersen I, Diderichsen F. Employment consequences of depressive symptoms and work demands individually and combined. Eur J Public Health 2014 Feb;24(1):34–9. https://doi.org/10.1093/eurpub/ckt011.

16. Oude Hengel K, Robroek SJ, Eekhout I, van der Beek AJ, Burdorf A. Educational inequalities in the impact of chronic diseases on exit from paid employment among older workers: a 7-year prospective study in the Netherlands. Occup Environ Med 2019 Oct;76(10):718–25. https://doi.org/10.1136/oemed-2019-105788.

17. Harber-Aschan L, Chen WH, McAllister A, Koitzsch Jensen N, Thielen K, Andersen I et al. The impact of longstanding illness and common mental disorder on competing employment exits routes in older working age: A longitudinal data-linkage study in Sweden. PLoS One 2020 Feb;15(2):e0229221. https://doi.org/10.1371/journal.pone.0229221.

18. Haddad RE, Matta J, Lemogne C, Melchior M, Zins M, Airagnes G. The association between substance use and subsequent employment among students: prospective findings from the CONSTANCES cohort. Soc Psychiatry Psychiatr Epidemiol 2023 Feb;58(2):249–66. https://doi.org/10.1007/s00127-022-02357-0.

19. Sundstrup E, Hansen AM, Mortensen EL, Poulsen OM, Clausen T, Rugulies R et al. Retrospectively assessed physical work environment during working life and risk of sickness absence and labour market exit among older workers. Occup Environ Med 2018 Feb;75(2):114–23. https://doi.org/10.1136/oemed-2016-104279.

20. Chen WH. Health and transitions into nonemployment and early retirement among older workers in Canada. Econ Hum Biol 2019 Dec;35:193–206. https://doi.org/10.1016/j.ehb.2019.06.001.

21. Leijten FR, de Wind A, van den Heuvel SG, Ybema JF, van der Beek AJ, Robroek SJ et al. The influence of chronic health problems and work-related factors on loss of paid

employment among older workers. J Epidemiol Community Health 2015 Nov;69(11):1058–65. https://doi.org/10.1136/jech-2015-205719.

22. Mastekaasa A. Unemployment and health: selection effects. J Community Appl Soc. 1996;6(3):189–205. https://doi.org/10.1002/(SICI)1099-1298(199608)6:3<189::AID-CASP366>3.0.CO;2-O.

23. Junna L, Moustgaard H, Martikainen P. Health-related selection into employment among the unemployed. BMC Public Health 2022 Apr;22(1):657. https://doi.org/10.1186/s12889-022-13023-0.

24. Moher D, Liberati A, Tetzlaff J, Altman DG, Group P. Preferred reporting items for systematic reviews and meta-analyses: the PRISMA statement. PLoS Med. 2009 Jul 21;6(7):e1000097. https://doi.org/10.1371/journal.pmed.1000097.

25. Armijo-Olivo S, Stiles CR, Hagen NA, Biondo PD, Cummings GG. Assessment of study quality for systematic reviews: a comparison of the Cochrane Collaboration Risk of Bias Tool and the Effective Public Health Practice Project Quality Assessment Tool: methodological research. J Eval Clin Pract 2012 Feb;18(1):12–8. https://doi.org/10.1111/j.1365-2753.2010.01516.x.

26. Thern E, Landberg J, Hemmingsson T. Educational differences in labor market marginalization among mature-aged working men: the contribution of early health behaviors, previous employment histories, and poor mental health. BMC Public Health 2020 Nov;20(1):1784. https://doi.org/10.1186/s12889-020-09899-5.

27. Carr E, Fleischmann M, Goldberg M, Kuh D, Murray ET, Stafford M et al. Occupational and educational inequalities in exit from employment at older ages: evidence from seven prospective cohorts. Occup Environ Med 2018 May;75(5):369–77. https://doi.org/10.1136/oemed-2017-104619.

28. Sewdas R, van der Beek AJ, Boot CR, D’Angelo S, Syddall HE, Palmer KT et al. Poor health, physical workload and occupational social class as determinants of health-related job loss: results from a prospective cohort study in the UK. BMJ Open 2019 Jul;9(7):e026423. https://doi.org/10.1136/bmjopen-2018-026423.

29. Rice NE, Lang IA, Henley W, Melzer D. Common health predictors of early retirement: findings from the English Longitudinal Study of Ageing. Age Ageing 2011 Jan;40(1):54–61. https://doi.org/10.1093/ageing/afq153.

30. Gong CH, He X. Factors Predicting Voluntary and Involuntary Workforce Transitions at Mature Ages: evidence from HILDA in Australia. Int J Environ Res Public Health 2019 Oct;16(19):3769. https://doi.org/10.3390/ijerph16193769.

31. Dong L, Agnew J, Mojtabai R, Surkan PJ, Spira AP. Insomnia as a predictor of job exit among middle-aged and older adults: results from the Health and Retirement Study. J Epidemiol Community Health 2017 Aug;71(8):750–7. https://doi.org/10.1136/jech-2016-208630.
32. Kagan R, Shiozawa A, Epstein AJ, Espinosa R. Impact of sleep disturbances on employment and work productivity among midlife women in the US SWAN database: a brief report. Menopause 2021 Aug;28(10):1176–80. https://doi.org/10.1097/GME.0000000000001834.
33. Sundstrup E, Thorsen SV, Rugulies R, Larsen M, Thomassen K, Andersen LL. Importance of the Working Environment for Early Retirement: Prospective Cohort Study with Register Follow-Up. Int J Environ Res Public Health 2021 Sep;18(18):9817. https://doi.org/10.3390/ijerph18189817.
34. Takada M, Tabuchi T, Iso H. Newly diagnosed disease and job loss: a nationwide longitudinal study among middle-aged Japanese. Occup Environ Med 2021 Apr;78(4):279–85. https://doi.org/10.1136/oemed-2020-106685.
35. Schinkel-Ivy A, Mosca I, Mansfield A. Factors Contributing to Unexpected Retirement and Unemployment in Adults Over 50 Years Old in Ireland. Gerontol Geriatr Med 2017 Jul;3:2333721417722709. https://doi.org/10.1177/2333721417722709.
36. Saarinen T, Savukoski SM, Pesonen P, Vaaramo E, Laitinen J, Varanka-Ruuska T et al. Climacteric status at age 46 is associated with poorer work ability, lower 2-year participation in working life, and a higher 7-year disability retirement rate: a Northern Finland Birth Cohort 1966 study. Menopause 2024 Apr;31(4):275–81. https://doi.org/10.1097/GME.0000000000002327.
37. Kujanpää L, Arffman RK, Vaaramo E, Rossi HR, Laitinen J, Morin-Papunen L et al. Women with polycystic ovary syndrome have poorer work ability and higher disability retirement rate at midlife: a Northern Finland Birth Cohort 1966 study. Eur J Endocrinol 2022 Aug;187(3):479–88. https://doi.org/10.1530/EJE-22-0027.
38. Rossi HR, Uimari O, Arffman R, Vaaramo E, Kujanpää L, Ala-Mursula L et al. The association of endometriosis with work ability and work life participation in late forties and lifelong disability retirement up till age 52: A Northern Finland Birth Cohort 1966 study. Acta Obstet Gynecol Scand 2021 Oct;100(10):1822–9. https://doi.org/10.1111/aogs.14210.
39. Carlsson E, Hemmingsson T, Landberg J, Burström B, Thern E. Do early life factors explain the educational differences in early labour market exit? A register-based cohort study. BMC Public Health 2023 Aug;23(1):1680. https://doi.org/10.1186/s12889-023-16626-3.
40. Pedersen J, Framke E, Thorsen SV, Sørensen K, Andersen MF, Rugulies R et al. The linkage of depressive and anxiety disorders with the expected labor market affiliation

(ELMA): a longitudinal multi-state study of Danish employees. Int Arch Occup Environ Health 2023 Jan;96(1):93–104. https://doi.org/10.1007/s00420-022-01906-z.

41. Pedersen J, Bjorner JB, Andersen LL. Physical work demands and expected labor market affiliation (ELMA): prospective cohort with register-follow-up among 46 169 employees. Scand J Work Environ Health 2022 Nov;48(8):641–50. https://doi.org/10.5271/sjweh.4050.

42. Pedersen J, Solovieva S, Thorsen SV, Andersen MF, Bültmann U. Expected Labor Market Affiliation: A New Method Illustrated by Estimating the Impact of Perceived Stress on Time in Work, Sickness Absence and Unemployment of 37,605 Danish Employees. Int J Environ Res Public Health 2021 May;18(9):4980. https://doi.org/10.3390/ijerph18094980.

43. Runge K, van Zon SK, Henkens K, Bültmann U. Metabolic syndrome increases the risk for premature employment exit: A longitudinal study among 60 427 middle-aged and older workers from the Lifelines Cohort Study and Biobank. Scand J Work Environ Health 2023 Nov;49(8):569–77. https://doi.org/10.5271/sjweh.4113.

44. Jennen JG, Jansen NW, van Amelsvoort LG, Slangen JJ, Kant I. Chronic conditions and self-perceived health among older employees in relation to indicators of labour participation and retirement over time. Work 2022;71(1):133–50. https://doi.org/10.3233/WOR-210436.

45. van de Ven D, Robroek SJ, Oude Hengel KM, Burdorf A, Schuring M. Changes in health among 45-64-year-old Dutch persons before, during and after becoming unemployed or employed: a seven year follow-up study. Scand J Work Environ Health 2022 May;48(4):283–92. https://doi.org/10.5271/sjweh.4016.

46. Wang M, Svedberg P, Narusyte J, Farrants K, Ropponen A. Effects of age on psychosocial working conditions and future labour market marginalisation: a cohort study of 56,867 Swedish twins. Int Arch Occup Environ Health 2022 Jan;95(1):199–211. https://doi.org/10.1007/s00420-021-01704-z.

47. De Breij S, Mäcken J, Qvist JY, Holman D, Hess M, Huisman M et al. Educational differences in the influence of health on early work exit among older workers. Occup Environ Med 2020 Aug;77(8):568–75. https://doi.org/10.1136/oemed-2019-106253.

48. Porru F, Burdorf A, Robroek SJ. The impact of depressive symptoms on exit from paid employment in Europe: a longitudinal study with 4 years follow-up. Eur J Public Health 2019 Feb;29(1):134–9. https://doi.org/10.1093/eurpub/cky136.

49. Reeuwijk KG, van Klaveren D, van Rijn RM, Burdorf A, Robroek SJ. The influence of poor health on competing exit routes from paid employment among older workers in 11 European countries. Scand J Work Environ Health 2017 Jan;43(1):24–33. https://doi.org/10.5271/sjweh.3601.

50. Kouwenhoven-Pasmooij TA, Burdorf A, Roos-Hesselink JW, Hunink MG, Robroek SJ. Cardiovascular disease, diabetes and early exit from paid employment in Europe; the impact of work-related factors. Int J Cardiol 2016 Jul;215:332–7. https://doi.org/10.1016/j.ijcard.2016.04.090.

51. Bethge M, Radoschewski FM, Gutenbrunner C. The Work Ability Index as a screening tool to identify the need for rehabilitation: longitudinal findings from the Second German Sociomedical Panel of Employees. J Rehabil Med 2012 Nov;44(11):980–7. https://doi.org/10.2340/16501977-1063.

52. Park S, Cho SI, Jang SN. Health conditions sensitive to retirement and job loss among Korean middle-aged and older adults. J Prev Med Public Health 2012 May;45(3):188–95. https://doi.org/10.3961/jpmph.2012.45.3.188.

53. Leino-Arjas P, Liira J, Mutanen P, Malmivaara A, Matikainen E. Predictors and consequences of unemployment among construction workers: prospective cohort study. BMJ 1999 Sep;319(7210):600–5. https://doi.org/10.1136/bmj.319.7210.600.

54. Carlsson E, Hemmingsson T, Almroth M, Falkstedt D, Kjellberg K, Thern E. Mediating effect of working conditions on the association between education and early labour market exit: a cohort study of Swedish men. Occup Environ Med 2024 Dec;81(11):547–55. https://doi.org/10.1136/oemed-2024-109594.

55. Levinson D, Kaplan G. What does Self Rated Mental Health Represent. J Public Health Res 2014 Dec;3(3):287. https://doi.org/10.4081/jphr.2014.287.

56. Lee JO, Jones TM, Yoon Y, Hackman DA, Yoo JP, Kosterman R. Young Adult Unemployment and Later Depression and Anxiety: Does Childhood Neighborhood Matter? J Youth Adolesc 2019 Jan;48(1):30–42. https://doi.org/10.1007/s10964-018-0957-8.

57. Lahelma E, Laaksonen M, Lallukka T, Martikainen P, Pietiläinen O, Saastamoinen P et al. Working conditions as risk factors for disability retirement: a longitudinal register linkage study. BMC Public Health 2012 Apr;12:309. https://doi.org/10.1186/1471-2458-12-309.

58. Mather L, Ropponen A, Mittendorfer-Rutz E, Narusyte J, Svedberg P. Health, work and demographic factors associated with a lower risk of work disability and unemployment in employees with lower back, neck and shoulder pain. BMC Musculoskelet Disord 2019 Dec;20(1):622. https://doi.org/10.1186/s12891-019-2999-9.

59. Amick BC 3rd, McDonough P, Chang H, Rogers WH, Pieper CF, Duncan G. Relationship between all-cause mortality and cumulative working life course psychosocial and physical exposures in the United States labor market from 1968 to 1992. Psychosom Med 2002;64(3):370–81. https://doi.org/10.1097/00006842-200205000-00002.

60. Borg V, Kristensen TS. Social class and self-rated health: can the gradient be explained by differences in life style or work environment? Soc Sci Med 2000 Oct;51(7):1019–30. https://doi.org/10.1016/S0277-9536(00)00011-3.

61. Schrijvers CT, van de Mheen HD, Stronks K, Mackenbach JP. Socioeconomic inequalities in health in the working population: the contribution of working conditions. Int J Epidemiol 1998 Dec;27(6):1011–8. https://doi.org/10.1093/ije/27.6.1011.

62. Robroek SJ, Reeuwijk KG, Hillier FC, Bambra CL, van Rijn RM, Burdorf A. The contribution of overweight, obesity, and lack of physical activity to exit from paid employment: a meta-analysis. Scand J Work Environ Health 2013 May;39(3):233–40. https://doi.org/10.5271/sjweh.3354.
